# Global consequences of dam‐induced river fragmentation on diadromous migrants: a systematic review and meta‐analysis

**DOI:** 10.1111/brv.70032

**Published:** 2025-05-07

**Authors:** Jeffery C. F. Chan, Billy Y. K. Lam, David Dudgeon, Jia Huan Liew

**Affiliations:** ^1^ School of Biological Sciences, The University of Hong Kong Pok Fu Lam Hong Kong; ^2^ Science Unit Lingnan University 8 Castle Peak Road Tuen Mun Hong Kong; ^3^ Research Group Neurobiology of Magnetoreception, Max Planck Institute for Neurobiology of Behaviour‐Caesar Ludwig‐Erhard‐Allee 2 Bonn 53175 Germany; ^4^ International Max Planck Research School for Brain and Behaviour Bonn Germany; ^5^ School of Natural Sciences, University of Tasmania Private Bag 51 Hobart Tasmania 7001 Australia

**Keywords:** diadromy, migratory, marine–freshwater, barrier, mitigation, fish pass, dam removal, fish, crustacean

## Abstract

The global proliferation of dams has altered flow and sediment regimes in rivers, presenting a major threat to freshwater biodiversity. Diadromous species, such as fishes, decapod crustaceans and gastropods, are particularly susceptible to fragmentation because dams obstruct their breeding migrations between coastal waters and rivers. Although dams have contributed to significant declines in abundance of some commercially important diadromous fishes (salmonids and anguillids) and *Macrobrachium* shrimps, understanding of the impacts of fragmentation on the majority of diadromous animals is limited. Moreover, the number of species known to have diadromous life cycles has risen substantially during the last four decades, from ~250 to more than 800. This synthesis aims to consolidate the global impacts of fragmentation on diadromous animals and highlight potential knowledge gaps. We identified 338 publications documenting the impacts of dams on diadromous fishes and decapods, but this was reduced to 65 publications after application of our strict selection criteria. Specifically, we only included studies that compared unfragmented (e.g. undammed) or restored (e.g. dams with fish passes) with fragmented (e.g. site above dams) rivers. To assess statistical significance, the results of studies that were replicated sufficiently to enable calculation of standardised effect sizes were also subject to meta‐analysis focusing on three topics: impacts of dam‐induced fragmentation; efficacy of fish passes; and the mitigative potential of dam removal. Study outcomes were evaluated from five key variables: abundance; species richness; assemblage composition; population genetic diversity; and population genetic structure. We found that fragmentation led to net negative effects across all key variables for diadromous fishes. Fishes with limited jumping or climbing ability and obligate diadromous migrants that cannot persist as landlocked populations were more threatened by fragmentation. However, fishes that were capable climbers or jumpers and facultatively diadromous were nonetheless susceptible to impacts, particularly in their abundance and gene flow between fragmented populations. Installation of fish passes did not lead to positive outcomes, whereas dam removal was effective in restoring connectivity for fishes, suggesting that it is a more effective, albeit potentially contentious, approach (e.g. the dam may serve an important societal need), for restoring habitat connectivity. A smaller number of publications investigated diadromous decapods (seven *versus* 61 on fishes), and our synthesis of their findings suggests that decapods were vulnerable to habitat alteration by dams, but were less sensitive to their barrier effects because they were better climbers than fishes. Gastropods were the least studied diadromous taxon, and none met our criteria for systematic review or meta‐analysis. The imbalance in information about diadromous taxa was compounded by a scarcity of studies from the tropics, particularly in South America, Africa, South Asia, Southeast Asia, and East Asia. These regions support diverse aquatic assemblages so the impacts of dams may be underestimated given existing knowledge gaps. The conservation of diadromous migrants would be best served by avoiding the construction of dams while improving mitigation strategies, such as fish passage design, to limit the most damaging effects of river fragmentation.

## I. INTRODUCTION

Dams represent a major threat to freshwater biodiversity (Turgeon, Turpin & Gregory‐Eaves, 2019; Barbarossa *et al*., 2020; Dudgeon & Strayer, 2024; He *et al*., 2024). Despite economic and societal benefits (e.g. hydropower, water and food security, and flood prevention), the construction of dams fragments rivers, causing significant habitat alteration and changes to flow and sediment regimes (Vörösmarty & Sahagian, 2000; Grill *et al*., 2015; Dudgeon, 2019). Fragmentation of freshwater habitats impacts freshwater species by impeding migratory routes to and from spawning and feeding grounds (Dudgeon, 2000; Verhelst *et al*., 2021). The impending expansion of hydropower facilities will further threaten freshwater biodiversity (Zarfl *et al*., 2015; Barbarossa *et al*., 2020). Around 90% of global river volume is already affected by fragmentation (Grill *et al*., 2015) and although this figure can be expected to increase it does not include the uncountable number of small dams globally.

Diadromous species are particularly vulnerable to dam‐induced fragmentation (henceforth ‘fragmentation’) because they migrate between coastal waters and rivers for reproduction (Verhelst *et al*., 2021). These migrants are geographically widespread, comprising mainly fishes (e.g. anguillid eels and salmonids), but also decapods (e.g. palaemonid shrimps such as *Macrobrachium* spp.), as well as a smaller number of gastropods (e.g. neritids such as *Clithon retropictum*). The number of species known to have diadromous life cycles has risen steeply from ~250 species of fishes in the 1990s (McDowall, 1992) to 444 fishes (Delgado & Ruzzante, 2020), 221 shrimps (de Mazancourt & Ravaux, 2024), and ~ 200 snails (Kano *et al*., 2011; Abdou, Keith & Galzin, 2015) at present. Many are commercially important, and underpin cultural practices and ecosystem services (Helfman, 2007; Limburg & Waldman, 2009). Diadromous species exhibit one of three distinct types of life cycles (McDowall, 2001): amphidromy (larvae drift to the sea from fresh water then return to rivers as juveniles), anadromy (adults migrate from the sea into rivers where they reproduce), and catadromy (adults migrate from rivers to reproduce in the sea). The proliferation of dams has contributed to a 73% decline in the abundance of 31% (138 species) of diadromous fishes since 1970 (Deinet *et al*., 2020); global records of reductions in the abundance of diadromous decapods or snails have yet to be compiled, although some impacts have been reported (e.g. Holmquist, Schmidt‐Gengenbach & Yoshioka, 1998; Ideguchi & Yamahira, 2004; Gorbach *et al*., 2012; Cooney & Kwak, 2013). In addition to presenting physical barriers to the movement of most fishes, dams also change in‐stream conditions with detrimental consequences (Reidy Liermann *et al*., 2012), including turbine‐strike mortality during downstream passage in hydropower installations (Montén, 1985; McLennan *et al*., 2018). Although some facultatively diadromous species can adapt and survive as landlocked populations above dams, they are genetically isolated from migratory populations that can exchange genetic material (Zarri *et al*., 2022).

More generally, the construction of dams has led to the collapse of commercial fisheries for diadromous species. Attempts to mitigate detrimental consequences of river fragmentation have involved the implementation of passage solutions, such as fish ladders, lifts, and traps (hereinafter referred to as fish passes). However, their effectiveness varies substantially according to species and migratory assemblage composition (Noonan, Grant & Jackson, 2012; Kemp, 2016; Silva *et al*., 2018), and the installation of fish passes designed for temperate species (mainly salmonids) in tropical rivers has not successfully mitigated the effects of barriers (Pompeu, Agostinho & Pelicice, 2012; Pelicice, Pompeu & Agostinho, 2015; Silva *et al*., 2018). By contrast, the removal of dams is considerably more effective, with the potential fully to restore migratory pathways and facilitate rapid recolonisation (reviewed by Waldman & Quinn, 2022). Nevertheless, understanding of the overall benefits of fish passes and dam removal on diadromous species is largely confined to salmonids, and a global synthesis of current knowledge is lacking, with most publications (e.g. Duarte *et al*., 2021) focusing on river fragmentation in north temperate latitudes; a notable exception is research from Puerto Rico (e.g. Cooney & Kwak, 2013).

In this study, we present the first comprehensive global synthesis of the impacts of dam‐induced fragmentation on diadromous species and the efficacy of measures to mitigate them. This study not only confirms existing assumptions about negative effects on diadromous species (i.e. dams cause negative impacts, fish passes are not always effective, dam removal is the most effective measure), but also identifies research gaps in terms of the taxonomic and geographic coverage of existing studies, while attempting to uncover some nuances of dam impacts (e.g. possible effects of life history or dam height), and provide a basis for future research. We undertook a systematic review of the literature with a focus on assessing the effects of dam‐induced fragmentation, the installation of fish passes, and dam removal on five key variables: abundance, richness, assemblage, genetic diversity, and genetic structure. The findings from each publication were categorised into one of three outcomes (negative, inconclusive, positive). To bolster the rigour of our evaluation, and following Borenstein *et al*. (2009) and Liew, Tan & Yeo (2016), we conducted a meta‐analysis that used Hedges' *g* effect sizes to quantify the statistical significance of a subset of publications that included replicated pairwise comparisons (for example) above and below dams or before and after dam removal for each diadromous species. Finally, the findings were summarised as a narrative synthesis to identify trends, vulnerable taxa and knowledge gaps to inform future research and the effective management of diadromous species in fragmented rivers.

## II. METHODS

### (1) Data collection

The structure of this study follows the guidelines stipulated by the Preferred Reporting Items for Systematic Reviews and Meta‐Analyses and its extension in ecology and evolutionary biology (PRISMA; O'Dea *et al*., 2021; Page *et al*., 2021; see online Supporting Information, Appendix S1 and Appendix S2). A comprehensive literature search was conducted using the *Web of Science* and *Scopus* scientific citation databases for papers published between January 1956 (*Web of Science*) or ‘before 1960’ (*Scopus*), and 20th June 2023. We used key words that yielded the most comprehensive list of publications for a representative sample of the literature (Table 1; see Appendix S1 for results of other search strings). The search terms were used to capture literature associated with five main themes: diadromy; fishes, decapods, and gastropods; biological metrics; dams and dam removal; and fish passes. One reviewer (J. C. F. C.) conducted a first‐pass eligibility assessment by scanning the title and abstract of each manuscript, followed by a second‐pass eligibility assessment of the full text (based on criteria set out in Table 2) by the same reviewer. Both eligibility assessments were screened by an additional reviewer (B. Y. K. L.).

**Table 1:** Key words that generated the greatest number of publications for review.

Category	Key words
Diadromy	‘Diadromous’ OR ‘amphidromous’ OR ‘anadromous’ OR ‘catadromous’ OR ‘migratory’
Fishes, decapods and gastropods	‘Fish’ OR ‘shrimp’ OR ‘prawn’ OR ‘gastropod’ OR ‘snail’ OR ‘crab’ OR ‘decapod’ OR ‘crustacean’
Biological metrics	‘Distribution’ OR ‘biomass’ OR ‘abundance’ OR ‘diversity’ OR ‘community’ OR ‘food web’ OR ‘assemblage’ OR ‘structure’ OR ‘genetic’ OR ‘trophic level’ OR ‘food chain length’ OR ‘trophic niche’
Dams and dam removal	‘Dam’ OR ‘obstructions’ OR ‘impoundment’ OR ‘reservoir’ OR ‘removal’
Fish passes	‘Fishway’ OR ‘fish pass’ OR ‘fish ladder’

**Table 2:** Eligibility criteria for inclusion in the systematic literature review (after Page *et al*., 2021).

First‐pass eligibility criteria (title, abstract, and publication venue)	Second‐pass eligibility criteria (full text)
Include: Organisms that are diadromous Publications on fragmentation or flow alteration by anthropogenic barriers Publications reporting empirical data Exclude: Non‐peer‐reviewed publications Exclusively modelling, behavioural, or review publications	Include publications that have a comparator of at least one of the following: Above and below dam Before and after damming Dammed reaches and undammed reaches Before and after dam removal Dams with and without fish passes Exclude studies exclusively on: Indices (e.g. Dendritic Connectivity Index) Estuarine habitats Hatchery and stocked fish

The review focused on three areas of interest: impacts of fragmentation, effectiveness of fish passes (including fish ladders, ramps, and various passage structures), and effects of dam removal. Overall, we found five major study designs across the three areas of interest. Publications on fragmentation included comparisons between (*i*) sites above *versus* sites below dams; (*ii*) dammed rivers *versus* undammed rivers; and (*iii*) sites before damming *versus* sites after damming. Studies of the effectiveness of fish passes involved comparison between (*iv*) dammed rivers without fish passes *versus* dammed rivers equipped with fish passes. Studies of the effects of dam removal involved comparisons between (*v*) sites before and after dam removal.

Valid comparisons will have a minimum of one control (i.e. a below‐dam site, an undammed river, a river before damming, a site before dam removal, or a dammed river without fish passes installed) and one ‘treatment’ data point (i.e. an above‐dam site, a dammed river, a river after damming, a site after dam removal, or a fishway‐equipped dammed river). In studies with more than one type of comparison (e.g. above *versus* below dams and dammed *versus* undammed rivers within the same publication), each comparison was assessed separately.

We extracted five key variables across comparisons (Table 3): abundance (AB); assemblage (AS); richness (RI); genetic diversity (GD); and genetic structure (GS). These variables were associated with different metrics, so we assessed them individually for every valid comparison. This means that a single publication may contain multiple outcomes for different key variables and/or metrics. For instance, comparisons between sites above and below dams from one river may yield outcomes for both abundance and richness or multiple metrics within abundance (e.g. density and biomass). Data were manually extracted from the main text, figures, tables, and supplementary data (when available) for each study, and can be found in Appendix S3 (also available at https://doi.org/10.5281/zenodo.14376862).

**Table 3:** Key variable for each target metric.

Key variable	Target metric used in publications
Abundance (AB)	Abundance, biomass, density
Assemblage (AS)	Community assemblage/structure, assemblage composition, proportion of diadromous species
Richness (RI)	Species richness, occurrence, spatial distribution
Genetic diversity (GD)	Allelic richness, expected heterozygosity
Genetic structure (GS)	Fixation index

### (2) Systematic review

Each key variable per valid comparison was summarised into one of three outcomes: positive; negative; inconclusive. This means that our total sample size (*N*), is number of publications × number of valid comparisons per publication × number of key variables per valid comparison.

We standardised outcomes across publications regardless of the number of species and/or number of sites in each to improve comparability. We outline specifics of how standardisation was applied across comparison types (e.g. comparing the abundance of one species between a pair of sites *vs* comparing the abundances of three species across a pair of sites) in Table 4, while Table 5 details how this was done for each of our three areas of interest. In general, a positive outcome was scored when the metrics of a treatment site(s) exceeded that of a control site(s), while a negative outcome reflected the opposite (Fig. S1). We categorised outcomes as inconclusive when there was no clear trend in a metric (e.g. abundance higher above dams for some species but lower for others) or if no statistically significant differences between treatment and control were reported (see Table 5). When appropriate, outcomes were also derived for metrics that were collected but not explicitly analysed in the primary source (e.g. retrieving richness data from a study that focused on abundance). Outcomes were scored according to the biodiversity implications of changes observed in each key variable (e.g. an increased fish population after damming represents a positive outcome for changes in abundance), regardless of the possible implications of underlying or indirect causes of the change (e.g. an increased fish population in response to a spike in primary productivity from nutrient loading would be scored as a positive outcome for abundance).

**Table 4:** Methods of assessing study outcomes across comparison types. All comparisons had a minimum of one pair of sites (one control and one treatment) for each species.

Comparison type	Methods
**Abbreviation: SS1** Single species in one pair of sites	Outcome is directly determined from the response of the species by comparing control against treatment sites.
**Abbreviation: SS2** Single species in more than two sites	Outcome is determined from the response across all sites and pooled to calculate a single outcome. If the number of control and treatment sites are uneven, the mean is calculated and compared to obtain the outcome. For publications using occurrence data, frequency of occurrence is used instead to determine the outcome of uneven comparisons.
**Abbreviation: MS** Multiple species in one pair of sites or in multiple sites	Individual outcome is assessed for each species using either of the methods above. Outcomes for all species are then pooled to determine the overall outcome of the publication based on the proportions of species that are positive, negative, or inconclusive.

**Table 5:** Criteria for determining study outcome under the three different areas of interest (i.e. fragmentation, fish passes, and dam removal).

Study outcome	Criteria
Positive	Fragmentation Target metric(s) are higher or statistically significant at treatment site (i.e. above dam/dammed river/river after damming) OR > 50% of treatment sites. Fish passes and dam removal Target metric(s) higher or statistically significant at treatment sites (i.e. river(s) with fish pass; river(s) after dam removal) OR > 50% of treatment sites. **Example 1:** 63% of sites recorded higher abundance of target species at treatment sites above the dam compared to control sites below the dam. **Example 2:** species richness was higher after dam removal compared to pre‐removal.
Negative	Fragmentation Target metric(s) lower or statistically significant in >50% of treatment sites (i.e. above dam/dammed river/after damming). Fish passes and dam removal Target metric(s) lower or statistically significant in >50% of treatment sites (i.e. rivers with fish pass; river after dam removal). **Example 1:** 75% of sites indicate higher abundance of target species at control sites below the dam than at treatment sites above the dam. **Example 2:** 96% of the pairwise fixation index F_ST_ comparisons between target species at control sites above the dam and at treatment sites below the dam were statistically significant.
Inconclusive	Either: (*a*) Target metric(s) not higher/lower or statistically significant >50% of control/ treatment sites. (*b*) For comparisons with multiple species in one pair of sites or in multiple sites, target metric(s) not higher/lower or statistically significant at control/treatment sites in >50% of species compared. (*c*) Results of statistical tests comparing target metric(s) between control and treatment were not significant. **Example A:** species assemblage was significantly different in 50% of sites after dam removal. **Example B:** in a study with four target species, two species were more abundant at the control site upstream of the dam, while the other two were more abundant below the dam. **Example C:** no significant difference in genetic differentiation (fixation index) between populations at treatment and control sites.

Where possible, the following information was also extracted from each of the publications included in the systematic review and meta‐analysis: (*i*) study design; (*ii*) study metric and response; (*iii*) dam height; (*iv*) list of diadromous species recorded in publication; and (*v*) region.

### (3) Meta‐analysis

To quantify and determine the statistical significance of the effects of dams and stream/river restoration efforts on diadromous animals, we calculated the bias‐corrected standardised mean differences using the ‘escalc’ function (Hedges' *g*; see below for methodology) (Borenstein *et al*., 2009; Liew *et al*., 2016; Viechtbauer, 2017). These calculations involved pairwise comparisons between control and treatment sites for each diadromous species in each publication across all metrics (Table 3). Hedges' *g* is a standardised effect size, which allowed us to compare outcomes quantitatively (i.e. mean differences) across studies. It represents the difference between two group means divided by the pooled standard deviation of those groups, and offers an accurate estimate of difference while adjusting for biases from small sample sizes (Borenstein *et al*., 2009). We further filtered publications from the systematic review, selecting only studies that had at least two control and two treatment sites for calculation of Hedges' *g* effect sizes. Each site was treated as one unit of replication for each species (e.g. a study of *Oncorhynchus mykiss* abundance in a single river system with three sites above and two sites below the dam would have three and two replicates, respectively). Surveys conducted at the same site across different periods were consolidated into a single mean value.

We conducted multilevel meta‐analytic models using the ‘rma.mv()’ function with restricted maximum‐likelihood estimation to account for the hierarchical structure arising from studies providing multiple effect sizes (Viechtbauer, 2017; Nakagawa *et al*., 2023b). To address variation among and between individual studies, we coded both study (i.e. publication or source of primary data) and individual effect sizes as random effects. We standardised effect size conventions across metrics so positive values indicate positive biodiversity outcomes (e.g. increased species richness, increased abundance). For example, greater abundance above dams or in dammed rivers was associated with positive Hedges' *g* values, while lower abundance after dam removal or implementation of fish passes resulted in negative Hedges' *g* scores. Through the use of Hedges' *g*, we were able to assess the impact of dams on different key variables. By pooling the effect sizes from different published findings, we can gain a concise and holistic view on the effects of dam impacts and river restoration. For each treatment, we calculated the *I* ^*2*^ index (Higgins & Thompson, 2002) and Q‐score (Huedo‐Medina *et al*., 2006) to detect and quantify respectively the degree of heterogeneity of unexplained variation between studies. High heterogeneity among studies suggests that the observed variation in effect sizes exceeds what would be expected by chance alone. This indicates significant differences in characteristics, methods, or results among the studies, which can offer insights into the factors contributing to their divergent results. To minimise the effects of substantial heterogeneity between studies (i.e. statistically significant Q‐score and *I* ^*2*^), we performed subgroup analyses using only studies reporting the same key variable (e.g. only abundance metrics). We then further evaluated heterogeneity between each subgroup of studies in order to assess whether valid conclusions could be drawn from comparisons yielding *I* ^*2*^ index and Q‐scores beyond recommended thresholds (Richardson, Garner & Donegan, 2019).

We assessed the reliability of meta‐analysis results by firstly constructing funnel plots to detect asymmetry in effect estimates (Sterne & Egger, 2001; Sterne & Harbord, 2004; Viechtbauer, 2017). Funnel‐plot asymmetry was tested where effect estimates included 10 or more studies, as the ability to identify true asymmetry is too low in instances with fewer studies (Sterne *et al*., 2011). To assess potential publication bias, we first tested for any small‐study effects whereby overall effect sizes are skewed by the disproportionally large influence of studies with low replication (Nakagawa *et al*., 2023b). Secondly, we tested for any decline effect, which tends to cause effect sizes to decrease over time because significant findings are more likely to be published earlier than non‐significant results (Nakagawa *et al*., 2023b). Finally, we conducted sensitivity analyses using a leave‐one‐out approach (Nakagawa *et al*., 2023b). This accounted for possible effects of geographic or phylogenetic non‐independence on meta‐analysis results by constructing a variance–covariance matrix, setting the correlation between observed effect sizes at 0.5 (Nakagawa *et al*., 2023b). Differences in effect sizes alone were not regarded as evidence of outlier‐driven biases, and meta‐analysis results were considered robust if the exclusion of any one study did not change the direction or statistical significance of effect sizes (Viechtbauer & Cheung, 2010; Viechtbauer, 2017). All analyses were conducted in the R (version 4.4.1) environment using the *metafor* and *orchard* packages (Viechtbauer, 2017; Nakagawa *et al*., 2023a).

## III. RESULTS

### (1) Overview

**Fig. 1:**
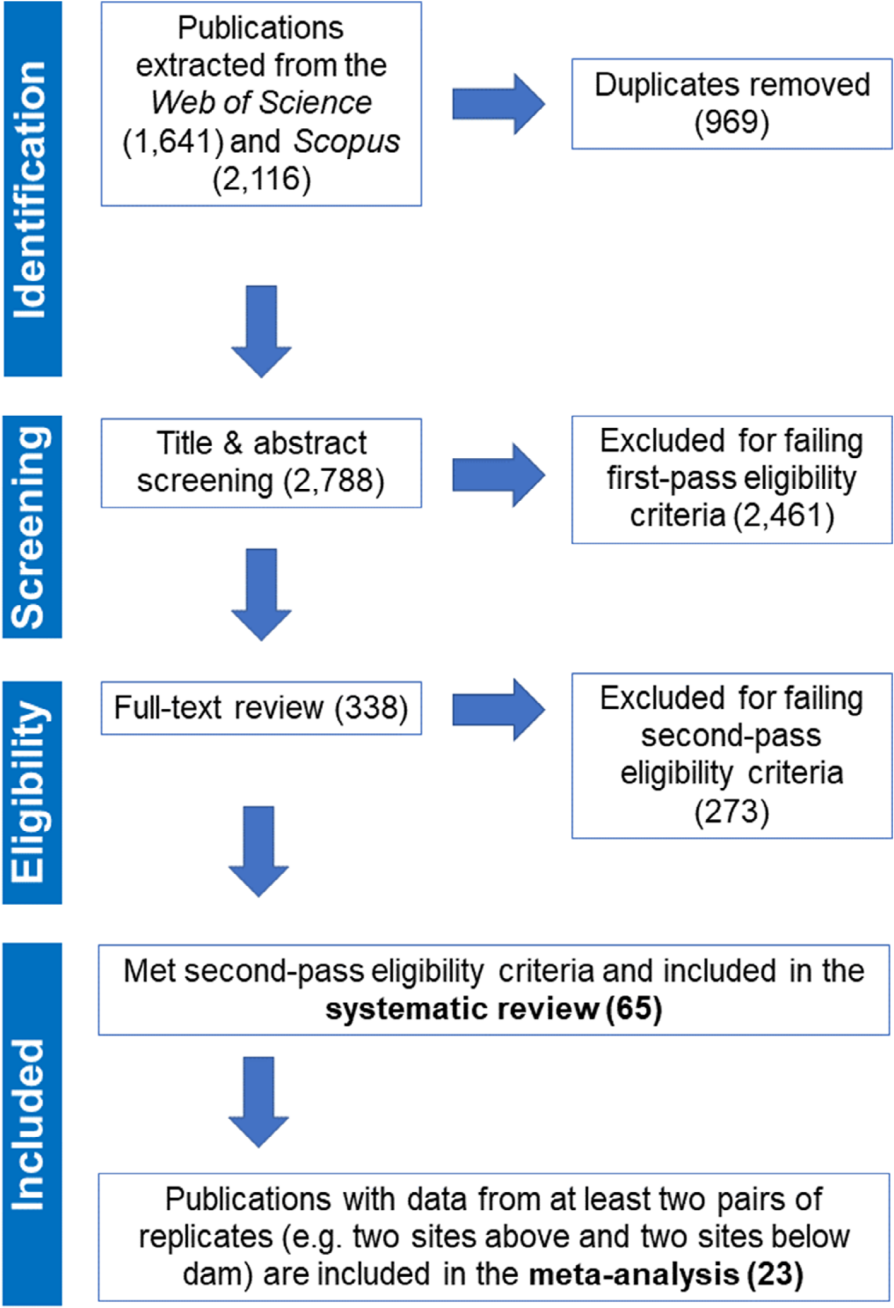
PRISMA flow diagram of the literature searching and screening process based on eligibility criteria in Table 2. Tallies reported in the figure are inclusive of studies involving fish and decapods.

A literature search that concluded on June 20, 2023 yielded 1,641 peer‐reviewed publications from *Web of Science* and 2,116 from *Scopus*. Of these, 338 publications met the first‐pass eligibility criteria (Table 2). Only 65 studies subsequently met the second‐pass eligibility criteria and were retained for review (Fig. 1). They comprised 58 studies that focused solely on fishes and four on decapods, while three publications included both fishes and decapods. No publications on gastropods met the criteria for review. A total of 114 outcomes were generated from the 65 publications from both taxa. Below, results for decapods are reviewed separately from fishes. Publications included both cross‐sectional and longitudinal study designs of varying durations; detailed metadata are available in Appendix S3.

### (2) Systematic review for fishes

#### (a) Impacts of dam‐induced fragmentation

**Fig. 2:**
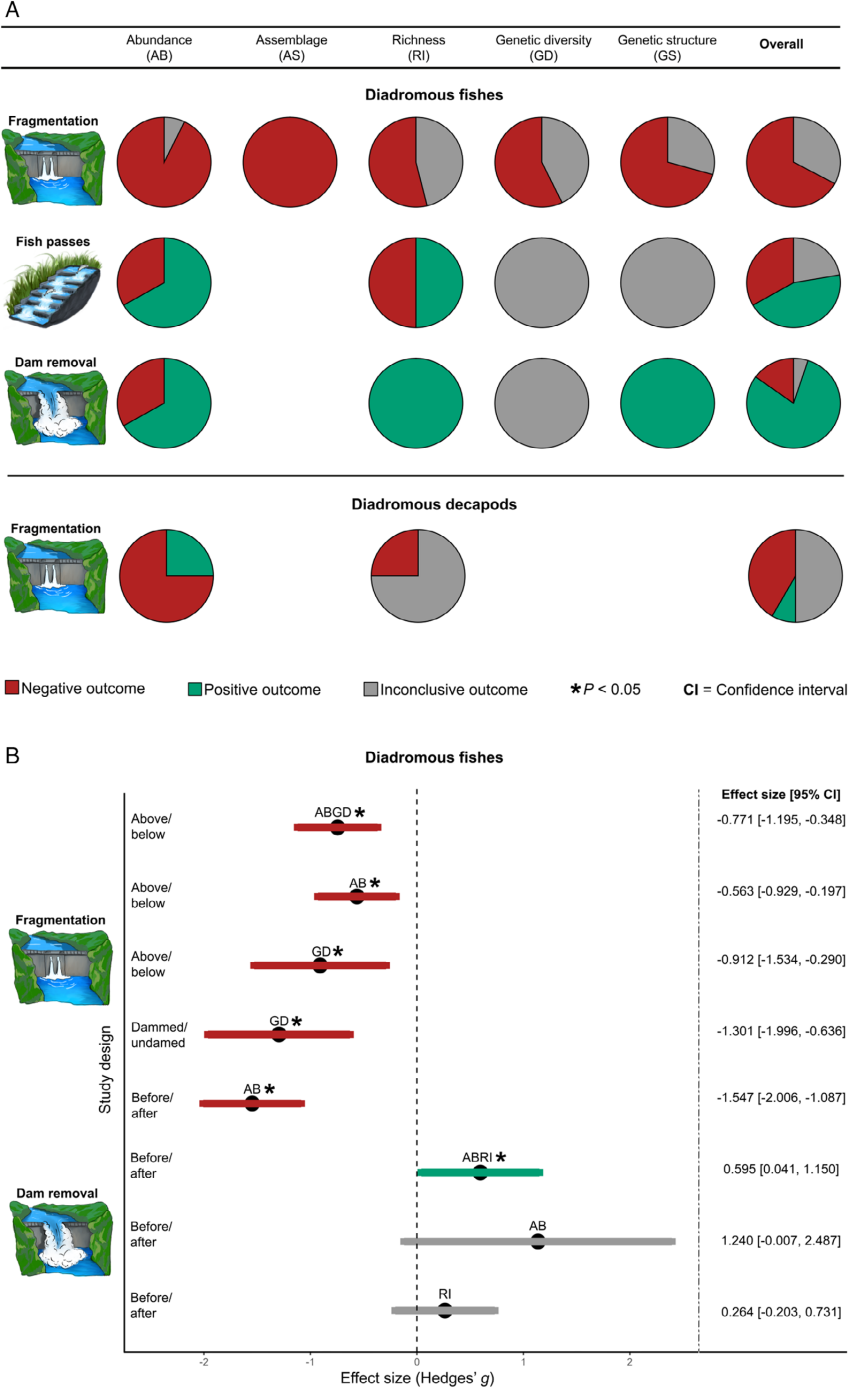
(A) Summary of outcomes in the systematic review of responses of diadromous fishes to impacts of dam‐induced fragmentation (*N* = 73), the implementation of fish passes (*N* = 9), and dam removal (*N* = 20) and diadromous decapods to dam‐induced fragmentation by dams (*N* = 12). (B) Summary forest plot of effect sizes in the meta‐analysis across different study designs (above *vs* below dams (ABGD = 42, AB = 26, GD = 16), dammed *vs* undammed rivers (GD = 6), before *vs* after damming (AB = 13), and before *vs* after dam removal (ABRI = 22, AB = 7, RI = 15); ABGD denotes pooled effect sizes of AB (second row) and GD (third row); ABRI denotes pooled effect size of AB (seventh row) and RI (eighth row); see Figs. S2–S7 for detailed forest plots and leave‐one‐out sensitivity analyses; ABGD, abundance and genetic diversity; AB, abundance; GD, genetic diversity; RI, richness.

A total of 73 outcomes on fragmentation were recorded from 40 publications. They were overwhelmingly negative (49 outcomes), although there were some (24) inconclusive outcomes (Fig. 2A; Table S1). Outcomes for assemblage composition were all negative, whereas 13 of 14 and 14 of 26 were negative for abundance and richness, respectively. Negative outcomes similarly were apparent for genetic attributes, affecting over half (eight of 14) of genetic diversity and the majority (12 of 17) of genetic structure comparisons.

A total of 52 outcomes were retrieved for comparisons of sites above and below dams, more than half (32) of which were negative and 20 inconclusive (Table S1). Outcomes were all negative for abundance and assemblage composition, but were far less consistent for richness (nine of 18 were negative). Although outcomes of genetic comparisons varied, the commonest outcomes for both genetic diversity (five of 11) and genetic structure (eight of 13) were negative.

In total, 13 outcomes were derived from publications with comparisons between dammed and undammed rivers, of which 11 were negative and two were inconclusive (Table S1). Specifically, outcomes involving abundance, genetic diversity, and genetic structure were all negative. Changes in richness were again more variable but were negative overall (three negative outcomes, two inconclusive). None of the studies meeting our criteria reported outcomes for assemblage composition.

Only eight outcomes were recorded from publications comparing rivers before and after damming, with the majority (six) reporting negative outcomes of dams (Table S1). All outcomes were negative for assemblage composition and the majority for abundance (three of four outcomes) and richness (two of three outcomes). None of the studies meeting our criteria reported outcomes for genetic diversity or structure.

#### (b) Fish‐pass efficacy

Although the literature describing the efficacy of fish passes is extensive, few studies have compared fishes in rivers with and without fish passes, and only nine outcomes from eight publications were found. Four outcomes showed positive effects of fish passes (two each for abundance and richness), whereas three (one abundance, two richness) reported negative effects and two (one each for genetic diversity and genetic structure) were inconclusive (Fig. 2A; Table S2).

#### (c) Dam removal

A total of 20 outcomes were recorded from 13 publications assessing the effects of dam removal. Outcomes were mostly positive (16 from abundance, richness and genetic structure), while negative and inconclusive outcomes were observed in three (abundance) and one (genetic diversity) comparisons, respectively (Fig. 2A; Table S3).

#### (d) Influence of dam height

Only 20 publications that focused on fragmentation impacts provided information about dam heights, although, in some of these cases, the data were only available online. A majority of these publications (12) dealt with large dams >15 m in height, whilst fewer (nine publications) covered small dams <15 m in height, and one publication included both large and small dams. Smaller dams were associated with more negative outcomes (14 of 19 outcomes from nine publications) than large dams (13 of 23 outcomes from 12 publications). All publications on fish passes that included information about dam height (four publications, five outcomes) focused on small dams, and returned an inconclusive outcome (two positive, one negative, and two inconclusive). Six of 10 publications on dam removal also focused on small dams, but the other four dealt with large dams. Overall positive outcomes were associated with the removal of both small (8 of 10 outcomes from six publications) and large dams (4 of 6 outcomes from four publications).

#### (e) Diversity and life history

Data on a total of a minimum of 118 diadromous fish species were represented across the 61 publications that contained information on fishes (Table S4), accounting for approximately 27% of the 444 known diadromous fishes (Delgado & Ruzzante, 2020). Publications often included species associated with more than one life‐history type: anadromy was best represented (40 publications), followed by catadromy (26) and amphidromy (14). Amphidromy and anadromy were represented by a total of 47 species each, accounting for 21% and 32% of known amphidromous and anadromous fishes, whereas the 24 catadromous species that were included comprised 33% of known catadromous fishes (Delgado & Ruzzante, 2020). The systematic review revealed a major imbalance in the coverage of amphidromous fish, with the majority of publications focusing on fragmentation; only two dealt with fish passes, and none reported responses to dam removal. Diadromous species were comparatively well served by publications concerning fragmentation (involving 106 species), followed by publications about the effects of dam removal (24 species) and effectiveness of fish passes (15 species). Of the 118 diadromous fishes included in the review, 11 were listed as threatened [Vulnerable (Vu), Endangered (EN) or Critically Endangered (CR)] by the IUCN (IUCN, 2023; Table S4).

#### (f) Regional data coverage

**Fig. 3:**
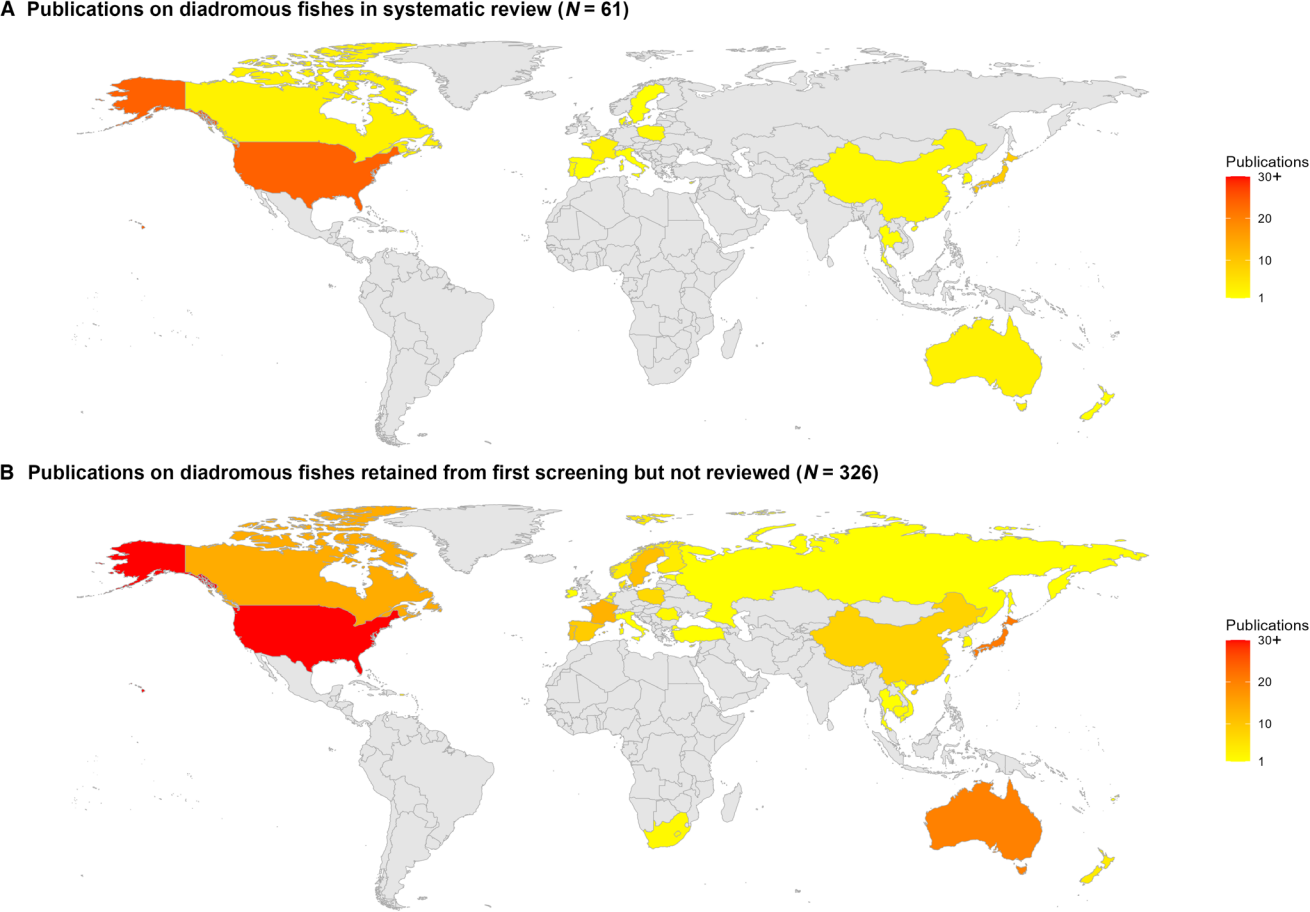
Number of publications on diadromous fishes from regions that were (A) included in systematic review and (B) are among the 265 publications that passed the first‐pass eligibility criteria (see Table 2) but failed to meet the second‐pass criteria for inclusion in the systematic review.

Six regions were represented across the 61 eligible publications on diadromous fishes: North America (27 publications), East Asia (i.e. Japan, China, and South Korea; 12), Europe (13), Oceania (five), the Caribbean (three), and one from Southeast Asia (Fig. 3A). No publications from Africa, South Asia, or South America met the eligibility criteria for review. This same pattern was evident from the 265 additional publications only on diadromous fishes that were screened, but not included in the review (see Fig. 3). Evidence of uneven research effort was also apparent in the composition of published studies meeting our search criteria, with more than half from the United States (46%) or Japan (22%).

### (3) Meta‐analysis for fishes

A total of 23 publications met our filtering criteria for meta‐analyses: 16 dealt with fragmentation and seven with dam removal. No publications on fish passes were suitable for analysis.

#### (a) Impacts of dam‐induced fragmentation

Our systematic search protocol yielded 16 publications covering 38 fish species. These consisted of 61 comparisons (i.e. effect size *N* = 61) across key variables describing trends in populations and genetic diversity (Fig. S2).

Pooled biodiversity outcomes, represented by abundance and genetic diversity (ABGD, Fig. 2B), were significantly higher below than above dams (pooled estimate = −0.771, *P* = < 0.001, 95% CI = −1.195, −0.348). The Q‐score was significant (*P* = 0.004), indicating potential heterogeneity, while the *I* ^*2*^ value suggested moderate heterogeneity (38.566%) in the pooled model. Subgroup analysis was therefore computed individually for abundance and genetic diversity of diadromous fishes. The results from both subgroups were consistent with the pooled model (Fig. 2B), signifying higher abundance (estimate = −0.563, P = 0.003, 95% CI = −0.929, −0.197) and genetic diversity (estimate = −0.912, *P* = 0.004, 95% CI = −1.534, −0.290) below dams, although the latter returned a statistically significant Q‐score (*P* = 0.004) when analysed separately from abundance. While visual inspection of the funnel plot across all comparisons between sites above and below dams indicated some asymmetry (Fig. S3), there was no evidence of any publication bias. Moreover, the moderate heterogeneity amongst studies, combined with leave‐one‐out analysis results, consistently showed higher abundance and genetic diversity below dams across iterations (Fig. S4), again implying that there was minimal publication bias, and confirming the robustness of our findings.

For comparisons between dammed and undammed rivers, genetic diversity was the only key variable available, and this was significantly higher in undammed rivers (Fig. 2B) (estimate = −1.301, *P* = < 0.001, 95% CI = −1.966, −0.636; Fig. S2). Both the Q‐score (*P* = 0.309) and *I* ^*2*^ value (20.961%) did not exceed the accepted thresholds for statistical heterogeneity, although the leave‐one‐out analysis returned a non‐significant effect size in one study (Harris *et al*., 2020), but directionality remained unchanged (Fig. S5). No publication bias was detected.

When comparing rivers before and after damming, abundance was the only key variable available. We used a random‐effects model to analyse effect sizes representing 13 species from a single study. Overall abundance was significantly higher before damming (Fig. 2B) (estimate = −1.547, *P* < 0.001, 95% CI = −2.006, −1.087; Fig. S2), and both the Q‐score and *I* ^*2*^ value were non‐significant (*P* = 0.229 and 20.023%, respectively); a leave‐one‐out analysis could not be conducted given that the results were derived from a single study.

#### (b) Dam removal

A total of seven publications dealing with 13 species yielded 22 counts of effect sizes measuring abundance and richness (see Fig. S6). The pooled effects of dam removal were positive and statistically significant (estimate = 0.596, *P* = 0.035, 95% CI = 0.041, 1.150; Figs 2B and S6), while both the Q‐scores and *I* ^*2*^ were non‐significant (*P* = 0.330 and 13.207%, respectively). Individually, however, both subgroup models for richness and abundance were non‐significant (richness estimate = 0.264, *P* = 0.269, 95% CI = −0.203, 0.731; abundance estimate = 1.240, *P* = 0.051, 95% CI = −0.007, 2.487), with abundance returning a statistically significant Q‐score (*P* = 0.047) as well as a broad 95% confidence interval (Fig. 2B). This uncertainty was reflected in the leave‐one‐out analysis, where more than half of the iterations returned non‐significant effect sizes, although all maintained the same directionality (Fig. S7). However, no publication bias was detected within this study design, and the low Q‐scores and *I* ^2^ between studies suggested that there was minimal bias in our findings that dam removal had positive effects on abundance and richness.

### (4) Systematic review for decapods

#### (a) Impacts of dam‐induced fragmentation

Only 12 outcomes were reported from the seven publications that dealt with decapods; all were focused on fragmentation. The outcomes were inconclusive overall, comprising five negative, six inconclusive, and one positive, respectively (Fig. 2A). The eight outcomes that compared decapods above and below dams yielded negative effects on abundance but inconclusive effects for richness (Table S5). By contrast, comparisons between dammed and undammed rivers were inconclusive: one positive and one negative outcome each for abundance and two inconclusive outcomes for richness (Table S5). No publications on decapods were suitable for meta‐analysis.

#### (b) Influence of dam height

Six of seven publications provided information on dam height. A majority of these publications (five) dealt with large dams >15 m in height, whilst two involved smaller dams, including one publication that had both large and small dams. There was no overall influence of dam height, although we note that half of the outcomes were negative for large dams compared to only one of five outcomes for small dams.

#### (c) Diversity and life history

A total of at least of 21 decapods from three families were included in the seven publications in the systematic review (Table S6): Atyidae (≥11 species); Palaemonidae (≥8); and Varunidae (two). This accounts for only 10% of the 221 known amphidromous shrimps (de Mazancourt & Ravaux, 2024) and an unknown percentage of the global diversity of diadromous crabs. All of the recorded decapods were amphidromous apart from the catadromous *Eriocheir japonica*. None of the decapods in Table S6 are considered to be threatened (IUCN, 2023).

#### (d) Regional data coverage

The seven publications containing information about decapods originated from East Asia (Japan, three publications), the Caribbean (two), Oceania (one), and North America (one). An additional eight publications that did not meet our second‐pass eligibility criteria included one publication each from two other regions (South America and Africa).

## IV. DISCUSSION

### (1) Effects of dam‐induced fragmentation on diadromous fishes

Our systematic review and meta‐analysis both arrived at the same overall conclusion: that dams have a negative impact on diadromous fishes. This applied across all variables studied, with reduced fish abundances, total species richness, and gene flow, along with significant changes in species assemblages.

The effects of dams on abundance were negative irrespective of fish climbing ability: even strong climbers such as *Anguilla* spp. (Shuai *et al*., 2021; Camhi *et al*., 2021) and *Sicydium* sp. (Frotté *et al*., 2020) were less abundant in reaches above dams. Facultatively diadromous fishes such as *Salmo trutta* (Consuegra *et al*., 2021), *Galaxias brevipinnis* (Gehrke, Gilligan & Barwick, 2002), *Tribolodon hakonensis* (Natsumeda & Seya, 2012), and *Percalates novemaculeata* (Rolls, 2011) were more abundant in dammed streams in some instances, probably due to their ability to transition to a primary freshwater life cycle. However, overall abundance of fishes in dammed rivers declined because the increase in facultatively diadromous fishes was more than offset by declines in obligate migrants, such as *Lampetra* sp. (Consuegra *et al*., 2021) and *Hypseleotris compressa* (Gehrke *et al*., 2002). There was only a single exception to the negative outcomes of damming among the studies we reviewed, with Azeiteiro *et al*. (2021) reporting a greater (although not significantly so) abundance of *Petromyzon marinus* in a dammed river. Our meta‐analysis results for fish abundance agreed with findings from the systematic review and, despite smaller sample sizes (four publications), the sensitivity and leave‐one‐out analyses confirmed that our results on the effects of dams were robust.

In comparison to abundance, the effects of dams on total species richness of fishes were less apparent, with more inconclusive outcomes (only one for abundance *versus* 12 for richness) despite being negative overall. This difference can be attributed to the fact that resilient species (i.e. strong climbers/jumpers or facultatively diadromous species such as salmonids) may avoid extirpation, yet suffer population declines. This is because individuals that manage to ascend dams incur higher energetic costs due to delays, heightened stress and increased immune system activity, which may affect fitness, resulting in reduced abundance above dams (Araújo, Ozório & Antunes, 2013; Rubenstein *et al*., 2022). For instance, *Anguilla* spp. occur above dams albeit at lower abundances than in an undammed tributary in an Australian river system (Rolls, 2011). The net effect on species richness was undetectable, because successful colonisation of upstream reaches by a single individual of each species resulted in equal richness above and below the obstacle. Accordingly, we suggest that, wherever possible in future studies, richness data should be supplemented by information on abundance to help uncover the contribution of remnant populations of resilient species to measures of overall richness.

The meta‐analysis outcomes for genetic diversity agreed with the findings of our systematic review, despite smaller sample sizes (11 *versus* 16 publications). The consistently lower levels of genetic diversity above dams are in accordance with a recent review that identified dam impacts on gene flow as major driver of reduced genetic diversity in river fishes (Zarri *et al*., 2022). Despite this, a relatively high proportion (43%) of the studies we reviewed had inconclusive genetic diversity outcomes in response to dams, possibly because they were mostly based on diadromous salmonids, alosids or gobiids that are often capable climbers or jumpers and, therefore, less susceptible to physical barriers. A single study (Harris *et al*., 2020) in the meta‐analysis of genetic diversity outcomes was particularly influential, and may have reflected frequent stocking of upstream populations with hatchery‐derived fish.

Systematic comparisons of the genetic structure of populations of diadromous and land‐locked fishes yielded significant differences in genetic differentiation (based on the fixation index *F*_*st*_) above and below dams (i.e. a negative outcome) in 12 of 17 publications, resulting from the lack of upstream gene flow. Salmonids (species of *Oncorhynchus* and *Salvelinus*) were particularly affected (negative outcomes in nine of 12 publications). In contrast to other life cycles, outcomes on amphidromous species (*Rhinogobius* sp. and *R. fluviatilis*) were inconclusive (Takagi *et al*., 2012, 2013). This is unlikely to be due to duration of fragmentation: for example, *R. fluviatilis* were landlocked for at least 35 years (Takagi *et al*., 2013), or approximately 35 generations (generation time ~ 1 year; Froese & Pauly, 2024); a comparable number of generations to 34 for *O. mykiss* (generation time ~ 3 years; Froese & Pauly, 2024) that had been landlocked for nearly a century in the Elwha River (DeHaan *et al*., 2011). One plausible explanation is that the fewer microsatellite loci used by Takagi *et al*. (2013) (3 *versus* 5–16 in other publications) and a lack of other markers (e.g. single nucleotide polymorphisms) may have limited the detection of genetic differentiation.

### (2) Effectiveness of fish passes

Our systematic review revealed that, overall, the effects of fish passes on species richness, genetic diversity and genetic structure, are mixed. This is consistent with a growing body of evidence on the relative ineffectiveness of fish passes (see Noonan *et al*., 2012; Kemp, 2016; Silva *et al*., 2018) which underscores a need for alternative solutions to restore migratory pathways. There are many issues that affect the success of fish passes. For instance, human disturbances (e.g. gravel extraction, pollution, dredging, intense fishing) have been associated with avoidance of fish passes (Santos *et al*., 2005). Fish passes may also act as ecological traps, attracting salmon from downstream spawning grounds into upstream reservoirs from where there are limited opportunities for them to return to natal spawning grounds (Twardek, Cooke & Lapointe, 2023). Furthermore, most fishes prefer to make nocturnal upstream movements (Jonsson, 1991), but manually controlled fish passes have locks that are only operational during the day thereby delaying migration and causing some individuals to stop moving upstream (Twardek *et al*., 2023). Even when fishes successfully ascend fish passes, they experience significant delays and incur energetic costs: *O. tshawytscha* took a median of 2.1 days to travel 366 m through a fish pass but could have moved 80 km upstream in a free‐flowing river during the same period (Twardek *et al*., 2023).

Fish passes were associated with higher abundance (two out of three publications). Numbers of *Anguilla anguilla* increased after the installation of specialised passes that enabled glass eels to ascend dams (Briand *et al*., 2005), while populations densities of both *O. kisutch* and *O. tshawytscha* were higher upstream of a dam one decade after installation of a fish pass (Kiffney *et al*., 2023). Salmonids also frequent fish passes, where they have a higher passage efficiency (62%) than non‐salmonid counterparts (21%; Noonan *et al*., 2012). Other fishes cannot ascend such structures, and small‐bodied species (<50 mm in length) such as *Gobiomorphus australis* failed to benefit from the fish‐pass installation in a subtropical Australian river (Rolls *et al*., 2018). In some instances, the design of fish passes is ineffective for the intended species. For instance, Tamario *et al*. (2019) found that, in Swedish rivers, the presence of specially designed narrow inclined ramps with bristles to help eels climb was negatively correlated with the probability of *A. anguilla* occurrence.

### (3) Effectiveness of dam removal for fishes

In contrast to fish passes, our findings revealed overall positive outcomes of dam removal across all key variables, with the exception of genetic diversity where differences were not detectible in the one publication in which it was documented. Relative to fish‐pass installation, dam removal achieved positive outcomes more quickly (between one and 11 years compared to 4–28 years), and may take place in as little as 5 days (ACFHP, 2017). Although our meta‐analysis revealed that dam removal had significantly positive overall effects, the sensitivity and leave‐one‐out analyses revealed some variability in the data, which is typical of meta‐analyses in ecology (Senior *et al*., 2016). This may have also been a consequence of the small sample sizes, and differences in study designs, the ways that dams were removed, or post‐removal monitoring. Nevertheless, we detected no small‐study effects, and minimal bias between studies.

Dam removals sometimes have negative biodiversity outcomes, contributing to variability in observations that may not have been evident from our systematic review criteria. For example, *Anguilla rostrata* biomass (but not abundance) decreased after dam removal, reflecting the influx of small individuals that formerly had been unable to pass the dam (Hitt, Eyler & Wofford, 2012). Unaddressed stressors may also be driving biodiversity loss (Haase *et al*. 2025): *Salmo salar* density decreased as fishes occupied sites upstream of the removed dam, and intense floods caused disturbance and sediment release that reduced the abundance of most species in the river (Magilligan *et al*., 2016). Nonetheless, our findings confirm the prevailing belief that dam removal is an effective restoration measure leading to rapid recovery of populations of diadromous fishes (Waldman & Quinn, 2022; Cancel Villamil & Locke, 2022), although some methodological improvements or standardisation of dam removal or monitoring protocols may help to reduce variability in success rates (e.g. Bastino, Boughaba & van de Bund, 2021; Schmidt, 2023). Significantly, dam removal does not depend on the ability of a fish to climb or jump, which can limit the success of fish passes.

Dam removal is not always feasible when dams are required for water storage, hydropower generation, and the provision of other societal benefits (Bednarek, 2001). Effective conservation may therefore require a mix of approaches (e.g. dam removal and fish passes), tailored to fit local needs (Waldman & Quinn, 2022). For instance, the Penobscot River Restoration Project in the USA removed two dams, improved four fish passes, and installed one new fish pass thereby allowing access to 3200 km of habitat upstream, while enhancing hydropower generation and increasing *Alosa pseudoharengus* migrations by a thousandfold (Opperman *et al*., 2011; Waldman & Quinn, 2022).

### (4) The effects of dam‐induced fragmentation on diadromous decapods

Our systematic review indicated that the effects of dams on decapods were inconclusive, apparently reflecting substantial differences in habitat preferences among species, but not disparities in climbing ability. Even decapods with differing habits (e.g. the hydrodynamically shaped *Atya* spp. *versus* benthic *Macrobrachium* spp.; Frotté *et al*., 2020) are able to ascend dams, and habitat generalists such as *Eriocheir japonica* can persist in dammed streams (Miya & Hamano, 1988; Ideguchi & Yamahira, 2004). Conversely, while *Caridina typus* and *C. leucosticta* can ascend dams through spillways, they do not thrive in impoundments with low flow rates (Ideguchi & Yamahira, 2004; Satake & Ueno, 2013) or where upstream habitat has been completely altered (Miya & Hamano, 1988). As was evident for diadromous fishes, decapod abundance was generally reduced by dams. Similarly, climbing probably incurs energetic costs for decapods, so that abundance above dams was reduced whereas colonisation success by a few individuals meant that species richness above and below dams did not differ.

Although some decapods are facultatively amphidromous (e.g. *Macrobrachium nipponense*; Chen *et al*., 2017), our literature review uncovered no publications on landlocked populations. Furthermore, there have been no studies on how the genetics of diadromous decapods are affected by dams. Since 45% of amphidromous atyids and 25% of *Macrobrachium* spp. are ‘Data Deficient’ or ‘Not Listed’ on the IUCN *Red List*, and a further 15% and 4% (respectively) have been categorised as threatened (IUCN, 2023; de Mazancourt & Ravaux, 2024), the potential impacts of dams on these animals warrants research attention.

### (5) Limitations

While our review was rigorous, the strict screening process may have excluded some study designs that might have uncovered other threats to diadromous animals. One example is the impacts of multiple dams, especially along river mainstems. For example, Brown *et al*. (2013) reported cumulative losses of *Salmo salar* and *Alosa sapidissima* across multiple dams in north‐eastern American rivers, despite the presence of fish passes, resulting in the exclusion of all but a few individuals from upstream spawning areas. In addition, we were unable to draw conclusions on how the characteristics of dams affect the barriers they present to movement. Undoubtedly, taller dams are more likely to prevent animal passage (Machut *et al*., 2007) and reduce species richness upstream (Chan, Liew & Dudgeon, 2024, 2025); other dam characteristics such as slope and surface texture may also be influential, as they are in the case of fish passes (Jellyman, Bauld & Crow, 2016), but were seldom reported in the studies included in our meta‐analysis.

Stocking rivers with commercial and game fishes is common in much of the northern hemisphere (and parts of the south), but this practice was not considered in our review. Dams may interact with the fish‐stocking practices if fish from hatcheries are concentrated within reaches above or below dams, where they cause genetic contamination of native diadromous species and reduce their fitness (Grant, 2012; Blouin *et al*., 2021). Examples include *O. mykiss* in the Willamette River, Oregon (Johnson *et al*., 2021), and *S. trutta* in a subarctic Pasvik River in Eurasia (Klütsch *et al*., 2019). Although such effects may have been underestimated in our meta‐analysis, they do not change the general conclusion that populations suffer genetic consequences as a result of dams.

Another consequence of our stringent screening criteria was the exclusion of a large number (45) of publications on fish passes from inclusion in the systematic review. Unfortunately, a large proportion of published fish pass research lacks comparisons with reference conditions (i.e. a dammed river without a fish pass, or population measurements made before fish‐pass installation), because of a historical focus on improving passage efficiency rather than achieving biodiversity benchmarks (Silva *et al*., 2018). This limited the quantitative assessments that could be made, although qualitative data on the efficacy of fish passes derived from our systematic review were generally similar to previous meta‐analyses of their efficiency (see Noonan *et al*., 2012; Kemp, 2016; Silva *et al*., 2018).

### (6) Knowledge gaps

One striking finding was that most published research on diadromous animals has been conducted in temperate North America, Europe, and Japan, with other regions that include freshwater biodiversity hotspots (South America and Africa, as well of parts of South, Southeast and East Asia) represented by few or no publications. The majority of diadromous animals, particularly those located in the tropics, are understudied, although many are likely subject to dam impacts since Asia and South America account for 48% of dams globally (Zhang & Gu, 2023). The scarcity of information from areas that represent current and future hotspots of dam construction, such as large portions of South Africa, South Asia, Southeast Asia and China (Winemiller *et al*., 2016; Barbarossa *et al*., 2020) persists, even when considering all 338 eligible articles (Fig. 3), including those that were ultimately unsuitable for review. This knowledge gap is aggravated by poor understanding of diadromous fish assemblages in these regions (McDowall, 2010), although recent studies have shown that diadromy is a frequently occurring life cycle in these regions (see Hauser *et al*., 2020; Vu *et al*., 2023; Chan *et al*., 2024, 2025). This is problematic because passage structures and management strategies developed in temperate areas are regarded as unsuitable for migratory assemblages in the tropics (Kemp, 2016; Silva *et al*., 2018).

The second obvious trend was an economic bias towards certain fishes. In part, this reflects the fact that the literature is dominated by studies of commercial species (i.e. salmonids, alosids, petromyzontids and anguillids); the same trend has been noted in reviews of fish‐pass effectiveness (see Noonan *et al*., 2012; Williams *et al*., 2012). Another notable deficiency was the relatively limited attention paid to amphidromous fishes, which are the most speciose group of diadromous fishes globally (Delgado & Ruzzante, 2020). Migratory decapods have received much less attention than their fish counterparts, and only 10% of known diadromous shrimps *versus* 27% of diadromous fish species (Delgado & Ruzzante, 2020; de Mazancourt & Ravaux, 2024) could be included in our systematic review. Aside from commercial species (i.e. *Macrobrachium. amazonicum*, *M. nipponense* and *M. rosenbergii*; de Mazancourt & Ravaux, 2024), little is known about the population ecology of most migratory decapods. Furthermore, estimates of the global richness of diadromous decapods is currently limited to amphidromous shrimps (221 species; de Mazancourt & Ravaux, 2024).

Gastropods have been the least researched group of diadromous animals, and there were insufficient publications to permit any systematic review or meta‐analysis of how they might be affected by dams. There are approximately 200 freshwater‐dwelling amphidromous gastropods globally, but little is known about their biology, ecology, or vulnerability to anthropogenic impacts (Kano *et al*., 2011; Abdou *et al*., 2015). What little information is available indicates that shear stress and streambed erosion associated with culverts impeded migration by amphidromous snails (*Neritina canalis*) in French Polynesia (Resh, Barnes & Craig, 1990; Resh, 2005), while *N. granosa* populations in Puerto Rico were similarly impacted by stream diversions (Gorbach *et al*., 2012).

The third general finding was a lack of publications containing information about bidirectional (i.e. upstream and downstream) passage. Most publications focused solely on upstream movement, and did not take account of downstream movements. The eggs, planktonic larvae, juveniles or migrating adults of amphidromous or potadromous fishes are more susceptible to fragmentation than catadromous species moving upstream (Pompeu *et al*., 2012; Pelicice *et al*., 2015; Jarvis & Closs, 2019). Similarly, downstream passage of some anadromous species is reduced by dams: only 10% of *Salmo salmar* smolts successfully migrated downstream in Winooski River (Vermont) despite the installation of fish passes (Nyqvist *et al*., 2017). In addition, turbine‐strike mortality (Montén, 1985; McLennan *et al*., 2018), as well as reduced environmental cues for migration and higher predation within impoundments (Marschall *et al*., 2011), can reduce or delay downstream passage.

## V. CONCLUSIONS

(1) Impacts of dam‐induced fragmentation on diadromous fishes were negative across all measurements. Fishes with limited jumping or climbing ability and obligate diadromous migrants that cannot persist as landlocked populations were more threatened by river fragmentation. Furthermore, the abundance of, and gene flow between populations of some facultatively diadromous fishes was reduced in the presence of dams, even among species that are relatively good at climbing or jumping.
(2) While fish passes can sometimes mitigate the obstruction of migration routes, their effectiveness varied with species. Good understanding of the composition of local diadromous assemblages and their behaviour, particularly in relation to climbing and jumping abilities, is necessary to maximise fish‐pass efficacy. In the absence of such information, fish passes should not be used as the primary or sole approach to mitigating dam impacts. Furthermore, reference conditions/sites should be established when evaluating fish passes to help assess if the desired biodiversity outcomes have been achieved by their installation.
(3) Where rivers have been fragmented by dams, their removal is an effective measure for restoring connectivity, and is likely to be effective without detailed knowledge of the composition of diadromous assemblages, and the behaviour of their component species.
(4) While dam removal can restore migratory pathways, the high costs of removal and the large number of dams fragmenting rivers globally, may limit the use of this conservation initiative. Furthermore, dam removal may be constrained by societal demand for the services they offer. There may also be some variability in its effectiveness across measured outcomes, but the implementation of standardised guidelines might reduce such variability through improvements in decommissioning methods, or detection of species in post‐removal monitoring surveys.
(5) Decapods appear to be more resistant to the impacts of fragmentation than fishes, possibly because they are relatively good climbers. They are nonetheless susceptible to habitat alterations due to damming; although the literature on this topic is scant and further research on potential impacts on their mitigation is needed. To date, very little is known about whether installation of fish passes or the removal of dams benefit migratory decapods.
(6) The full extent of dam impacts on diadromous species is likely to have been underestimated at the global scale due to limited research in the tropics, and the relative scarcity of studies on diadromous decapods or gastropods, and amphidromous fishes. The information reviewed herein reveals a need to mitigate the adverse consequences of dams on diadromous species and underscores the extent of variability in fish‐pass efficacy when such structures have been installed. Dam removal is a comparatively more effective way of mitigating the effects of river fragmentation than installing fish passes, but variable outcomes revealed by the meta‐analyses suggest that there might be confounding issues requiring additional studies to provide a more conclusive assessment. Avoiding dam construction wherever possible and improvements in mitigation measures (e.g. by enhancing accessibility of fish passes to small‐bodied species) are priority measures to limit the damaging effects of river fragmentation on diadromous animals.
(7) Finally, we advocate standardisation of protocols and study designs, including improved reporting of dam characteristics, to enhance the scope for understanding the consequences of river fragmentation on freshwater biodiversity.

## AUTHOR CONTRIBUTIONS

J. C. F. C., D. D. and J. H. L. conceived the research concept and study design. J. C. F. C. and B. Y. K. L. carried out data collection, data analysis, and drafting, while D. D. and J. H. L. contributed to writing, editing and supervision.

## Supporting information


**Appendix S1.** Selected key word searches and unselected search results.
**Table S1.** Outcomes of each study design across key variables from our systematic review of the impacts of dam‐induced fragmentation on diadromous fishes.
**Table S2.** Outcomes of each study design across key variables from the systematic review for diadromous fishes with comparisons on fish passes.
**Table S3.** Outcomes of each study design across key variables from the systematic review for diadromous fishes with comparisons on dam removal.
**Table S4.** Diadromous fishes recorded from 61 publications included in the systematic review.
**Table S5.** Outcomes of each study design across key variables from the systematic review for dam‐induced fragmentation on diadromous decapods.
**Table S6.** Diadromous decapods recorded from the seven publications included in the systematic review.
**Fig. S1.** Flowchart of systematic review workflow.
**Fig. S2.** Individual effect sizes for each species per publication on dam‐induced fragmentation included in the meta‐analysis.
**Fig. S3.** Funnel plots for the publications included in the meta‐analysis that used above/below dam study designs to investigate dam‐induced fragmentation.
**Fig. S4.** Leave‐one‐out analysis for studies included in the meta‐analysis that employed an above/below study design to investigate dam‐induced fragmentation.
**Fig. S5.** Leave‐one‐out analysis for studies included in the meta‐analysis that employed a dammed/undammed study design to investigate dam‐induced fragmentation.
**Fig. S6.** Individual effect sizes for each species in each publication on dam removal included in the meta‐analysis.
**Fig. S7.** Leave‐one‐out analysis for studies included in the meta‐analysis that investigated dam removal.

**Appendix S2.** PRISMA eco‐evo checklist.

**Appendix S3.** Excel spreadsheet(s) of publications that underwent screening, full text review, and subsequent inclusion in the systematic review and meta‐analysis.
