# Supplementary material for: Global consequences of dam‐induced river fragmentation on diadromous migrants: a systematic review and meta‐analysis
Source: Biol Rev Camb Philos Soc. 2025 May 7;100(5):2020–37. doi: 10.1111/brv.70032 (PMC12407043; doi:10.1111/brv.70032)
Supplement: Supplementary file 1 — Appendix S1. Selected key word searches and unselected search results. Table S1. Outcomes of each study design across key variables from our systematic review of the impacts of dam‐induced fragmentation on diadromous fishes. Table S2. Outcomes of each study design across key variables from the systematic review for diadromous fishes with comparisons on fish passes. Table S3. Outcomes of each study design across key variables from the systematic review for diadromous fishes with comparisons on dam removal. Table S4. Diadromous fishes recorded from 61 publications included in the systematic review. Table S5. Outcomes of each study design across key variables from the systematic review for dam‐induced fragmentation on diadromous decapods. Table S6. Diadromous decapods recorded from the seven publications included in the systematic review. Fig. S1. Flowchart of systematic review workflow. Fig. S2. Individual effect sizes for each species per publication on dam‐induced fragmentation included in the meta‐analysis. Fig. S3. Funnel plots for the publications included in the meta‐analysis that used above/below dam study designs to investigate dam‐induced fragmentation. Fig. S4. Leave‐one‐out analysis for studies included in the meta‐analysis that employed an above/below study design to investigate dam‐induced fragmentation. Fig. S5. Leave‐one‐out analysis for studies included in the meta‐analysis that employed a dammed/undammed study design to investigate dam‐induced fragmentation. Fig. S6. Individual effect sizes for each species in each publication on dam removal included in the meta‐analysis. Fig. S7. Leave‐one‐out analysis for studies included in the meta‐analysis that investigated dam removal. [file BRV-100-2020-s001.docx]

**Appendix S1.**

**The selected keyword search yielded the most publications and was used for analyses. Unselected keyword searches yielded fewer publications and were not selected for analyses.**

**Selected** keyword search (1956 – 20/06/2023) *Web of Science*, (before 1960 – 20/06/2023) *Scopus*

*Web of Science* – **1,641 articles**, *Scopus* – **2,116 articles**

("diadromous" OR "amphidromous" OR "anadromous" OR "catadromous" OR “migratory”) AND ("dam" OR "obstructions" OR “impoundment” OR “reservoir” OR "fishway" OR "fish pass" OR "fish ladder" OR “removal”) AND (“fish” OR “shrimp” OR “prawn” OR “gastropod” OR “snail” OR “crab” OR “decapod” OR “crustacean” OR “distribution” OR "biomass" OR "abundance" OR "diversity" OR "community" OR "food web" OR “assemblage” OR “structure” OR “genetic” OR “trophic level” OR “food chain length” OR “trophic niche”)

Results of unselected keyword searches (1956 – 20/06/2023) *Web of Science*, (before 1960 – 20/06/2023) *Scopus*

1. *Web of Science* – **607 articles**, *Scopus* – **936 articles**

("diadromous" OR "amphidromous" OR "anadromous" OR "catadromous" OR “migratory”) AND ("dam" OR "obstructions" OR “impoundment” OR "fishway" OR "fish pass" OR "fish ladder" OR “removal”) AND ("distribution" OR "biomass" OR "abundance" OR "diversity" OR "community" OR "food web" OR “assemblage” OR “structure”) AND (“fish” OR “invertebrate” OR “crustacean” OR “crab” OR “shrimp” OR “prawn” OR “snail”)

1. *Web of Science* – **1,641 articles**, *Scopus* – **2,113 articles**

("diadromous" OR "amphidromous" OR "anadromous" OR "catadromous" OR “migratory”) AND ("dam" OR "obstructions" OR “impoundment” OR “reservoir” OR "fishway" OR "fish pass" OR "fish ladder" OR “removal”) AND (“fish” OR “shrimp” OR “gastropod” OR “snail” OR “crab” OR “decapod” OR “distribution” OR "biomass" OR "abundance" OR "diversity" OR "community" OR "food web" OR “assemblage” OR “structure” OR “genetic” OR “trophic level” OR “food chain length” OR “trophic niche”)

1. *Web of Science* – **1,641 articles**, *Scopus* – **2,115 articles**

("diadromous" OR "amphidromous" OR "anadromous" OR "catadromous" OR “migratory”) AND ("dam" OR "obstructions" OR “impoundment” OR “reservoir” OR "fishway" OR "fish pass" OR "fish ladder" OR “removal”) AND (“fish” OR “shrimp” OR “gastropod” OR “snail” OR “crab” OR “decapod” OR “crustacean” OR “distribution” OR "biomass" OR "abundance" OR "diversity" OR "community" OR "food web" OR “assemblage” OR “structure” OR “genetic” OR “trophic level” OR “food chain length” OR “trophic niche”)

**Table S1.** Outcomes of each study design across key variables from our systematic review of the impacts of dam-induced fragmentation on diadromous fishes.

| **Above/below dams** | **Negative** | **Positive** | **Inconclusive** | **Sub–total** |
| --- | --- | --- | --- | --- |
| Abundance (AB) | 9 | – | – | 9 |
| Assemblage (AS) | 1 | – | – | 1 |
| Richness (RI) | 9 | – | 9 | 18 |
| Genetic diversity (GD) | 5 | – | 6 | 11 |
| Genetic structure (GS) | 8 | – | 5 | 13 |
| **Dammed/undammed** |  |  |  |  |
| Abundance (AB) | 1 | – | – | 1 |
| Richness (RI) | 3 | – | 2 | 5 |
| Genetic diversity (GD) | 3 | – | – | 3 |
| Genetic structure (GS) | 4 | – | – | 4 |
| **Before/after damming** |  |  |  |  |
| Abundance (AB) | 3 | – | 1 | 4 |
| Assemblage (AS) | 1 | – | – | 1 |
| Richness (RI) | 2 | – | 1 | 3 |
| **Total** | **49** |  | **24** | **73** |

**Table S2.** Outcomes of each study design across key variables from the systematic review for diadromous fishes with comparisons on fish passes.

| **Fish pass** | **Negative** | **Positive** | **Inconclusive** | **Sub–total** |
| --- | --- | --- | --- | --- |
| Abundance (AB) | 1 | 2 | – | **3** |
| Richness (RI) | 2 | 2 | – | **4** |
| Genetic diversity (GD) | – | – | 1 | **1** |
| Genetic structure (GS) | – | – | 1 | **1** |
| **Total** | **3** | **4** | **2** | **9** |

**Table S3.** Outcomes of each study design across key variables from the systematic review for diadromous fishes with comparisons on dam removal.

| **Dam removal** | **Negative** | **Positive** | **Inconclusive** | **Sub–total** |
| --- | --- | --- | --- | --- |
| Abundance (AB) | 3 | 6 | – | **9** |
| Richness (RI) | – | 9 | – | **9** |
| Genetic diversity (GD) | – | – | 1 | **1** |
| Genetic structure (GS) | – | 1 | – | **1** |
| **Total** | **3** | **16** | **1** | **20** |

**Table S4.** Diadromous fishes recorded from the 61 publications included in the systematic review. IUCN status based on IUCN (2023): NE = not evaluated; LC = least concern; NT = near threatened; VU = vulnerable; EN = endangered; CR = critically endangered. * indicates facultatively diadromous species.

| **Order** | **Family** | **Species** | **Life history** | **IUCN** | **Fragmentation** | **Fish passes** | **Dam removal** |
| --- | --- | --- | --- | --- | --- | --- | --- |
| Acanthuriformes | Leiognathidae | *Leiognathus equula* | Amphidromous* | LC | Y |  |  |
|  | Scatophagidae | *Scatophagus argus* | Amphidromous* | LC | Y |  |  |
| Acropomatiformes | Lateolabracidae | *Lateolabrax japonicus* | Catadromous* | LC | Y |  |  |
| Anguilliformes | Anguillidae | *Anguilla anguilla* | Catadromous | CR | Y | Y |  |
|  | Anguillidae | *An. australis* | Catadromous | NT | Y |  |  |
|  | Anguillidae | *An. dieffenbachii* | Catadromous | EN | Y |  |  |
|  | Anguillidae | *An. japonica* | Catadromous | EN | Y |  |  |
|  | Anguillidae | *An. marmorata* | Catadromous | LC | Y |  |  |
|  | Anguillidae | *An. reinhardtii* | Catadromous | LC | Y | Y |  |
|  | Anguillidae | *An. rostrata* | Catadromous | EN | Y |  | Y |
| Beloniformes | Hemiramphidae | *Hyporhamphus intermedius* | Amphidromous* | NE | Y |  |  |
|  | Hemiramphidae | *Hy. sajori* | Amphidromous* | NE | Y |  |  |
| Carangaria *incertae sedis* | Latidae | *Lates calcarifer* | Catadromous* | LC | Y |  |  |
|  | Polynemidae | *Eleutheronema tetradactylum* | Amphidromous* | NE | Y |  |  |
| Centrarchiformes | Kuhliidae | *Kuhlia rupestris* | Catadromous | LC | Y |  |  |
|  | Percalatidae | *Percalates colonorum* | Catadromous* | LC | Y |  |  |
|  | Percalatidae | *Pe. novemaculeatus* | Catadromous* | NE | Y | Y |  |
| Clupeiformes | Alosidae | *Alosa aestivalis* | Anadromous* | VU | Y | Y | Y |
|  | Alosidae | *Al. alosa* | Anadromous* | LC | Y |  |  |
|  | Alosidae | *Al. fallax* | Anadromous* | LC | Y |  |  |
|  | Alosidae | *Al. mediocris* | Anadromous* | LC | Y |  | Y |
|  | Alosidae | *Al. pseudoharengus* | Anadromous* | LC | Y | Y | Y |
|  | Alosidae | *Al. sapidissima* | Anadromous* | LC |  |  | Y |
|  | Alosidae | *Al.* sp. | Anadromous* | NE | Y |  |  |
|  | Clupeidae | *Potamalosa richmondia* | Catadromous* | LC | Y |  |  |
|  | Dorosomatidae | *Anodontostoma chacunda* | Anadromous* | LC | Y |  |  |
|  | Dorosomatidae | *Escualosa thoracata* | Amphidromous* | LC | Y |  |  |
|  | Dorosomatidae | *Hilsa kelee* | Anadromous* | LC | Y |  |  |
|  | Engraulidae | *Coilia nasus* | Anadromous* | EN | Y |  |  |
|  | Engraulidae | *Coilia grayii* | Anadromous* | LC | Y |  |  |
| Cypriniformes | Leuciscidae | *Pseudaspius hakonensis* | Anadromous* | LC | Y |  |  |
|  | Leuciscidae | *Vimba vimba* | Anadromous* | LC | Y |  |  |
| Elopiformes | Megalopidae | *Megalops cyprinoides* | Amphidromous | DD | Y | Y |  |
| Eupercaria incertae sedis | Moronidae | *Morone americana* | Anadromous* | LC |  |  | Y |
|  | Moronidae | *Mo. saxatilis* | Anadromous* | LC | Y |  | Y |
| Galaxiiformes | Galaxiidae | *Galaxias argenteus* | Amphidromous* | VU | Y |  |  |
|  | Galaxiidae | *Ga. brevipinnis* | Amphidromous* | LC | Y |  |  |
|  | Galaxiidae | *Ga. fasciatus* | Amphidromous* | LC | Y |  |  |
|  | Galaxiidae | *Ga. maculatus* | Catadromous* | LC | Y |  |  |
|  | Galaxiidae | *Ga. postvectis* | Amphidromous* | EN | Y |  |  |
| Gobiiformes | Eleotridae | *Gobiomorphus australis* | Amphidromous* | LC | Y | Y |  |
|  | Eleotridae | *Go. cotidianus* | Amphidromous* | LC | Y |  |  |
|  | Eleotridae | *Go. coxii* | Amphidromous* | LC | Y |  |  |
|  | Eleotridae | *Go. gobioides* | Amphidromous* | LC | Y |  |  |
|  | Eleotridae | *Go. hubbsi* | Amphidromous* | VU | Y |  |  |
|  | Eleotridae | *Go. huttoni* | Amphidromous* | NT | Y |  |  |
|  | Eleotridae | *Hypseleotris compressa* | Amphidromous* | LC | Y |  |  |
|  | Gobiidae | *Bathygobius fuscus* | Amphidromous* | LC | Y |  |  |
|  | Gobiidae | *Favonigobius gymnauchen* | Amphidromous* | LC | Y |  |  |
|  | Oxudercidae | *Acanthogobius flavimanus* | Amphidromous* | LC | Y |  |  |
|  | Oxudercidae | *Ac. hasta* | Amphidromous* | LC | Y |  |  |
|  | Oxudercidae | *Ac. lactipes* | Amphidromous* | LC | Y |  |  |
|  | Oxudercidae | *Awaous guamensis* | Amphidromous | LC | Y |  |  |
|  | Oxudercidae | *Gymnogobius castaneus* | Amphidromous* | LC | Y |  |  |
|  | Oxudercidae | *Gy. petschiliensis* | Amphidromous* | LC | Y |  |  |
|  | Oxudercidae | *Gy. urotaenia* | Amphidromous* | LC | Y |  |  |
|  | Oxudercidae | *Gy.* spp. | Amphidromous* | NE | Y |  |  |
|  | Oxudercidae | *Leucopsarion petersii* | Amphidromous* | LC | Y |  |  |
|  | Oxudercidae | *Luciogobius guttatus* | Amphidromous* | LC | Y |  |  |
|  | Oxudercidae | *Periophthalmus modestus* | Amphidromous* | NE | Y |  |  |
|  | Oxudercidae | *Rhinogobius brunneus* | Amphidromous | DD | Y |  |  |
|  | Oxudercidae | *Rh. fluviatilis* | Amphidromous* | NE | Y |  |  |
|  | Oxudercidae | *Rh. kurodai* | Amphidromous | NE | Y |  |  |
|  | Oxudercidae | *Rh.* sp. CB | Amphidromous* | NE | Y |  |  |
|  | Oxudercidae | *Rh.* spp*.* | Amphidromous | NE | Y |  |  |
|  | Oxudercidae | *Sicydium plumieri* | Amphidromous | DD | Y |  |  |
|  | Oxudercidae | *Sicyd. punctatum* | Amphidromous | LC | Y |  |  |
|  | Oxudercidae | *Sicyd.* spp. | Amphidromous | NE | Y |  |  |
|  | Oxudercidae | *Smilosicyopus lepurus* | Amphidromous | NE | Y |  |  |
|  | Oxudercidae | *Stiphodon elegans* | Amphidromous | LC | Y |  |  |
|  | Oxudercidae | *Tridentiger brevispinis* | Amphidromous* | LC | Y |  |  |
|  | Oxudercidae | *Tr. obscurus* | Amphidromous* | LC | Y |  |  |
| Carangiformes | Pleuronectidae | *Platichthys flesus* | Catadromous* | LC | Y |  |  |
| Mugiliformes | Mugilidae | *Mugil cephalus* | Catadromous* | LC | Y | Y |  |
|  | Mugilidae | *Planiliza haematocheilus* | Catadromous* | NE | Y |  |  |
|  | Mugilidae | *Trachystoma petardi* | Catadromous* | LC | Y | Y |  |
| Osmeriformes | Osmeridae | *Hypomesus olidus* | Anadromous* | LC | Y |  |  |
|  | Osmeridae | *Osmerus mordax* | Anadromous* | LC | Y |  |  |
|  | Plecoglossidae | *Plecoglossus altivelis* | Amphidromous* | DD | Y |  |  |
|  | Retropinnidae | *Prototroctes maraena* | Amphidromous* | VU | Y |  |  |
|  | Retropinnidae | *Retropinna retropinna* | Anadromous* | LC | Y |  |  |
|  | Retropinnidae | *Stokellia anisodon* | Anadromous* | LC | Y |  |  |
|  | Salangidae | *Neosalanx andersoni* | Anadromous* | NE | Y |  |  |
|  | Salangidae | *Ne. jordani* | Anadromous* | LC | Y |  |  |
|  | Salangidae | *Salangichthys microdon* | Anadromous* | LC | Y |  |  |
| Perciformes | Cheimarrichthyidae | *Cheimarrichthys fosteri* | Amphidromous* | NE | Y |  |  |
|  | Cottidae | *Cottus aleuticus* | Catadromous* | LC |  |  | Y |
|  | Cottidae | *Co. asper* | Catadromous* | LC |  |  | Y |
|  | Cottidae | *Co. kazika* | Catadromous* | LC | Y |  |  |
|  | Cottidae | *Trachidermus fasciatus* | Catadromous* | NE | Y |  |  |
|  | Gasterosteidae | *Gasterosteus aculeatus* | Anadromous* | LC | Y |  |  |
|  | Tetrarogidae | *Notesthes robusta* | Catadromous* | LC | Y | Y |  |
| Petromyzontiformes | Geotriidae | *Geotria australis* | Anadromous | DD | Y |  |  |
|  | Mordaciidae | *Mordacia mordax* | Anadromous | LC | Y |  |  |
|  | Petromyzontidae | *Entosphenus tridentatus* | Anadromous | LC |  |  | Y |
|  | Petromyzontidae | *Lampetra.* sp. | Anadromous* | NE | Y |  |  |
|  | Petromyzontidae | *Lethenteron camtschaticum* | Anadromous* | LC | Y |  |  |
|  | Petromyzontidae | *Petromyzon marinus* | Anadromous* | LC | Y | Y | Y |
| Pleuronectiformes | Rhombosoleidae | *Rhombosolea retiaria* | Catadromous* | DD | Y |  |  |
| Salmoniformes | Salmonidae | *Oncorhynchus clarkii* | Anadromous* | NE |  |  | Y |
|  | Salmonidae | *On. gorbuscha* | Anadromous* | NE |  |  | Y |
|  | Salmonidae | *On. keta* | Anadromous* | NE | Y |  | Y |
|  | Salmonidae | *On. kisutch* | Anadromous* | NE |  | Y | Y |
|  | Salmonidae | *On. masou masou* | Anadromous* | NE | Y |  | Y |
|  | Salmonidae | *On. mykiss* | Anadromous* | NE | Y | Y | Y |
|  | Salmonidae | *On. nerka* | Anadromous* | LC | Y |  | Y |
|  | Salmonidae | *On. tshawytscha* | Anadromous* | NE |  | Y | Y |
|  | Salmonidae | *On.* sp. | Anadromous* | NE | Y |  |  |
|  | Salmonidae | *Salmo salar* | Anadromous* | NT | Y |  | Y |
|  | Salmonidae | *Sa. trutta* | Anadromous* | LC | Y | Y |  |
|  | Salmonidae | *Salvelinus confluentus* | Anadromous* | VU | Y |  | Y |
|  | Salmonidae | *Sal. fontalis* | Anadromous* | NE |  |  | Y |
|  | Salmonidae | *Sal. leucomaenis* | Anadromous* | NE | Y |  |  |
|  | Salmonidae | *Thymallus thymallus* | Anadromous* | LC | Y |  |  |
| Gadiformes | Gadidae | *Microgadus tomcod* | Anadromous* | LC |  |  | Y |
| Siluriformes | Bagridae | *Mystus gulio* | Anadromous* | LC | Y |  |  |
|  | Plotosidae | *Plotosus canius* | Amphidromous* | NE | Y |  |  |
| Acipenseriformes | Acipenseridae | Acipenseridae spp*.* | Anadromous | NE |  |  | Y |
| **Total number of species** |  |  |  |  | **106** | **15** | **24** |

**Table S5.** Outcomes of each study design across key variables from the systematic review for dam-induced fragmentation on diadromous decapods.

|  | **Negative** | **Inconclusive** | **Positive** | **Sub-total** |
| --- | --- | --- | --- | --- |
| **Above/below** |  |  |  |  |
| Abundance | 2 | - | - | **2** |
| Richness | 2 | 4 | - | **6** |
| **Dammed/undammed** |  |  |  |  |
| Abundance | 1 | - | 1 | **2** |
| Richness | - | 2 | - | **2** |
| **Total** | **5** | **6** | **1** | **12** |

**Table S6.** Diadromous decapods recorded from the seven publications included in the systematic review.

| **Family** | **Species** | **Life history** |
| --- | --- | --- |
| Atyidae | *Atya innocous* | Amphidromous |
|  | *At. scabra* | Amphidromous |
|  | *Atya* spp. | Amphidromous |
|  | *Micratya poeyi* | Amphidromous |
|  | *Xiphocaris elongata* | Amphidromous |
|  | *Caridina celebensis* | Amphidromous |
|  | *Ca. leucosticta* | Amphidromous |
|  | *Ca. japonica* | Amphidromous |
|  | *Ca. serratirostris* | Amphidromous |
|  | *Ca. typus* | Amphidromous |
|  | *Caridina* spp. | Amphidromous |
| Palaemonidae | *Macrobrachium faustinum* | Amphidromous |
|  | *Ma. formosense* | Amphidromous |
|  | *Ma. heterochirus* | Amphidromous |
|  | *Ma. japonicum* | Amphidromous |
|  | *Ma. lar* | Amphidromous |
|  | *Ma. nipponense* | Amphidromous |
|  | *Ma. ohione* | Amphidromous |
|  | *Macrobrachium* spp. | Amphidromous |
| Varunidae | *Eriocheir japonica* | Catadromous |
|  | *Ptychognathus glaber* | Amphidromous |


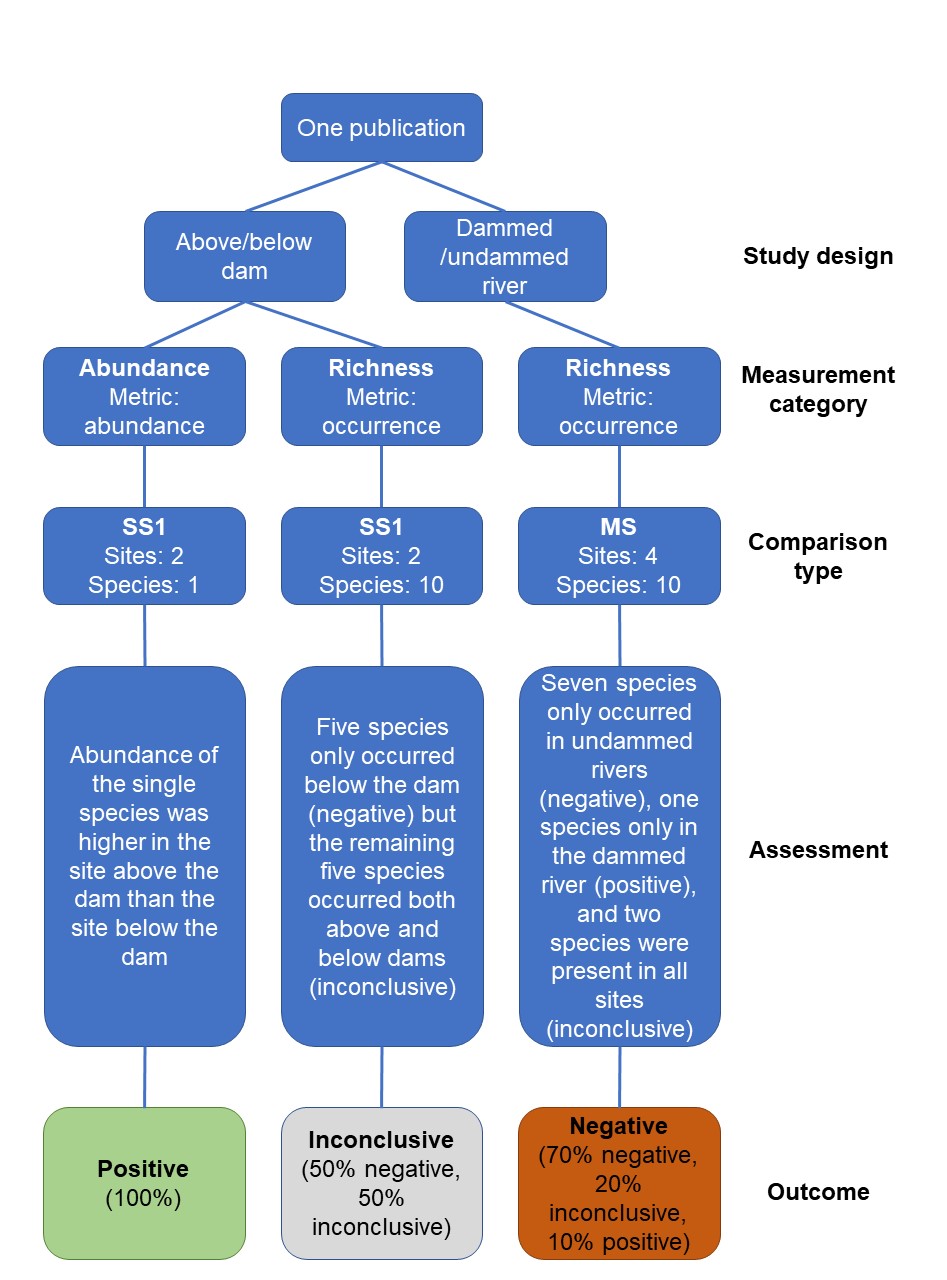
Fig. S1. Flowchart of systematic review workflow for a hypothetical publication with three outcomes. SS1 = single species in one pair of sites, MS = multiple species in one pair of sites or in multiple sites.

**
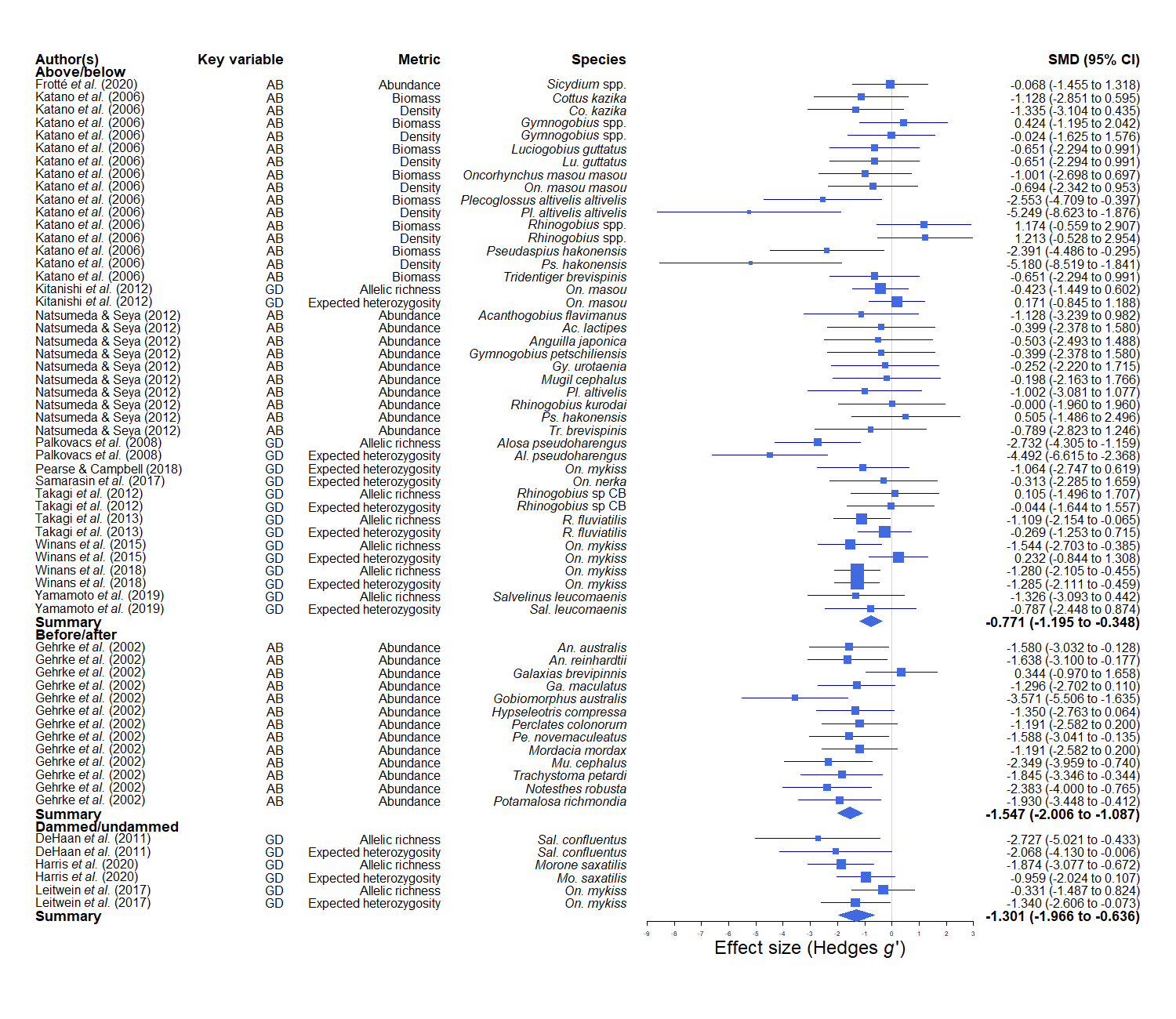
Fig. S2.** Individual effect sizes for each species per publication on dam-induced fragmentation included in the meta-analysis. Three study designs are represented: (1) above/below (sites above *versus* below dams), (2) before/after (sites before *versus* after damming), and (3) dammed/undammed (dammed *versus* undammed rivers). The key variable indicates the category of each metric used in each study, where AB represents abundance and GD represents genetic diversity. Effect sizes are expressed as the standardised mean difference (SMD), calculated as Hedges’ *g*. Square sizes correspond to the 95% confidence interval (CI), with larger squares indicating smaller CIs and lower variability across studies. Diamonds represent the overall effect size for each study design, summarising all individual effect sizes within that category.


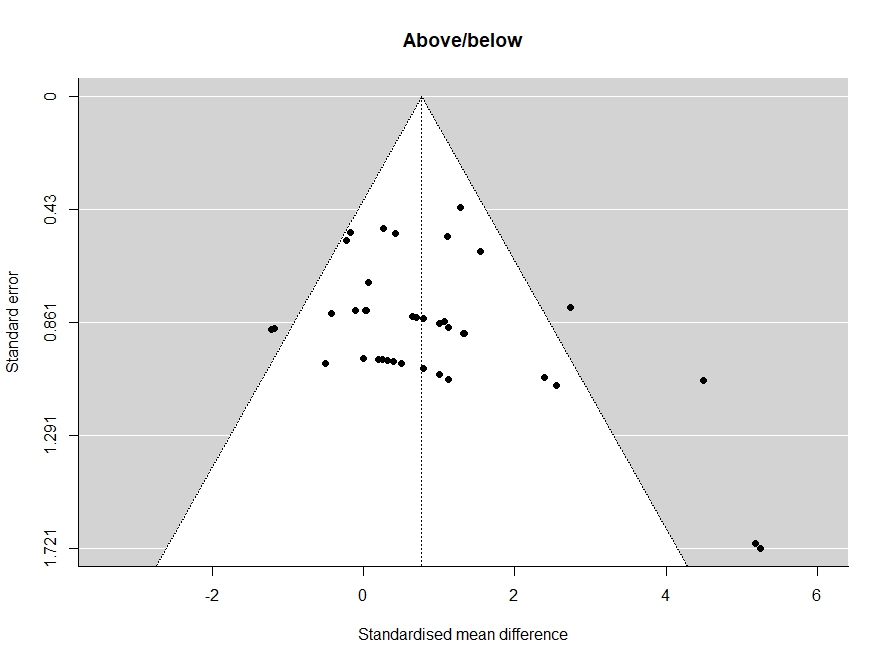
**Fig. S3.** Funnel plot for the publications included in the meta-analysis that used above/below dam study designs to investigate dam-induced fragmentation.

**
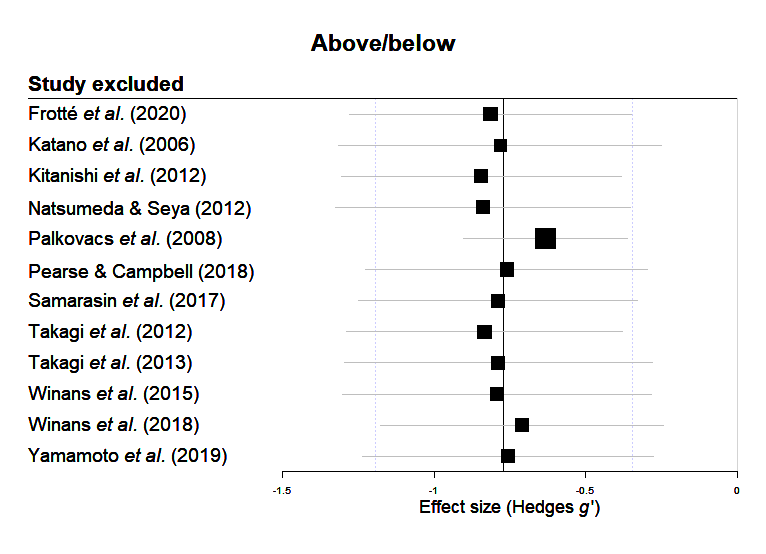
**

**Fig. S4.** Leave-one-out analysis of above/below study design on dam-induced fragmentation. Each row represents the overall effect size with the corresponding study removed. Square sizes indicate the 95% confidence interval (CI), with larger squares representing smaller CIs and lower variability across studies. The solid vertical line denotes the overall effect size when all studies are included, while the dashed lines represent its CI. If the horizontal CI line of an effect size crosses zero after removing a study, the effect becomes non-significant.


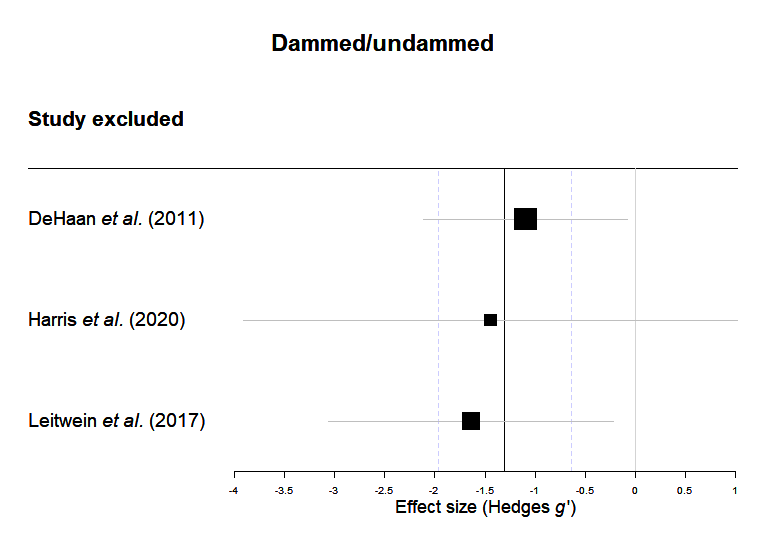


**Fig. S5.** Leave-one-out analysis for studies included in the meta-analysis that employed a dammed/undammed study design to investigate dam-induced fragmentation. Each row represents the overall effect size with the corresponding study removed. Square sizes indicate the 95% confidence interval (CI), with larger squares representing smaller CIs and lower variability across studies. The solid vertical line denotes the overall effect size when all studies are included, while the dashed lines represent its CI. If the horizontal CI line of an effect size crosses zero after removing a study, the effect becomes non-significant.


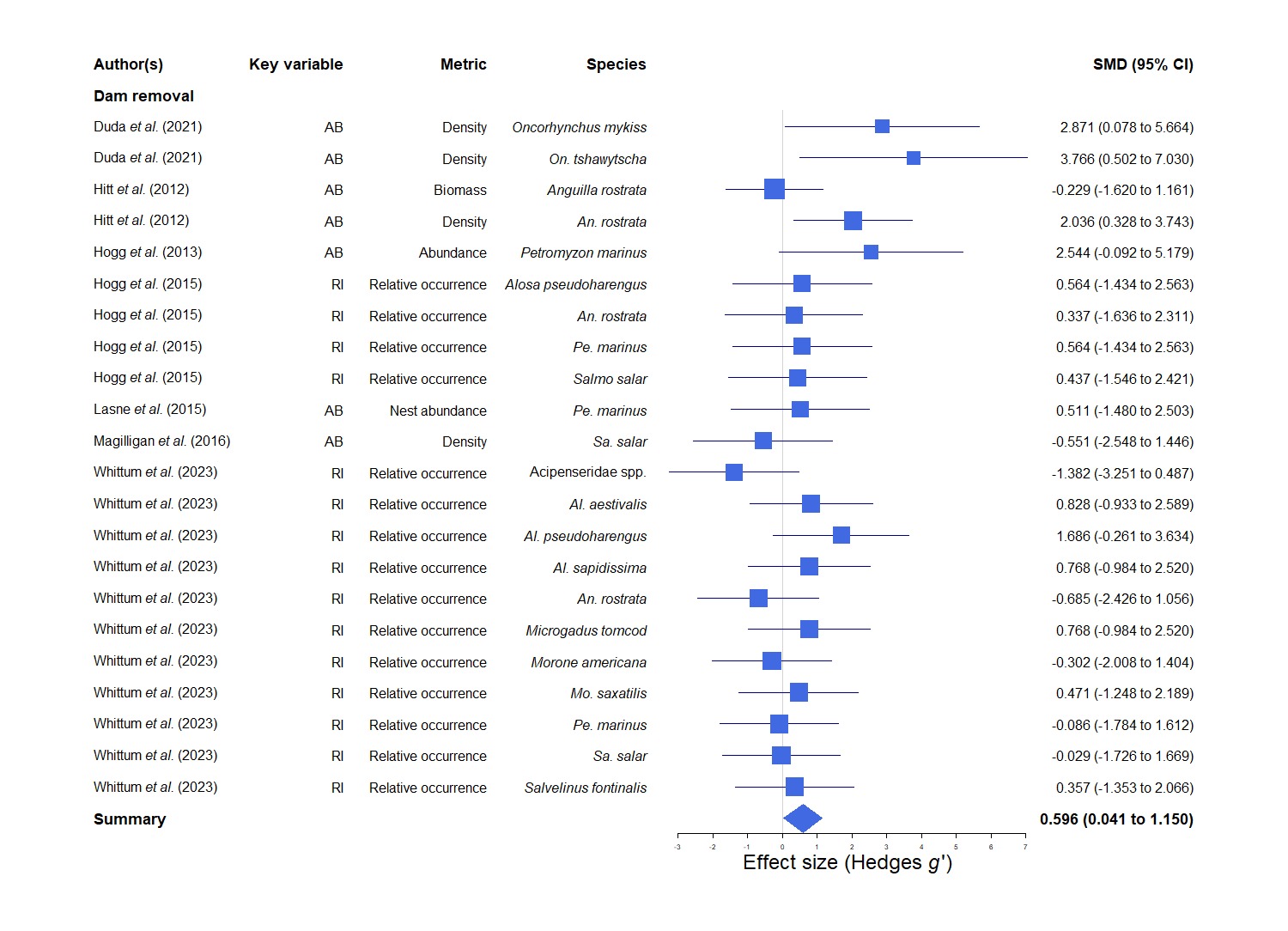
**Fig. S6.** Individual effect sizes for each species in each publication on dam removal included in the meta-analysis. The key variable indicates the category of each metric used in each study, where AB represents abundance and RI represents richness. Effect sizes are expressed as the standardised mean difference (SMD), calculated as Hedges’ *g*. Square sizes correspond to the 95% confidence interval (CI), with larger squares indicating smaller CIs and lower variability across studies. Diamonds represent the overall effect size for each study design, summarising all individual effect sizes within the dam removal category.

**
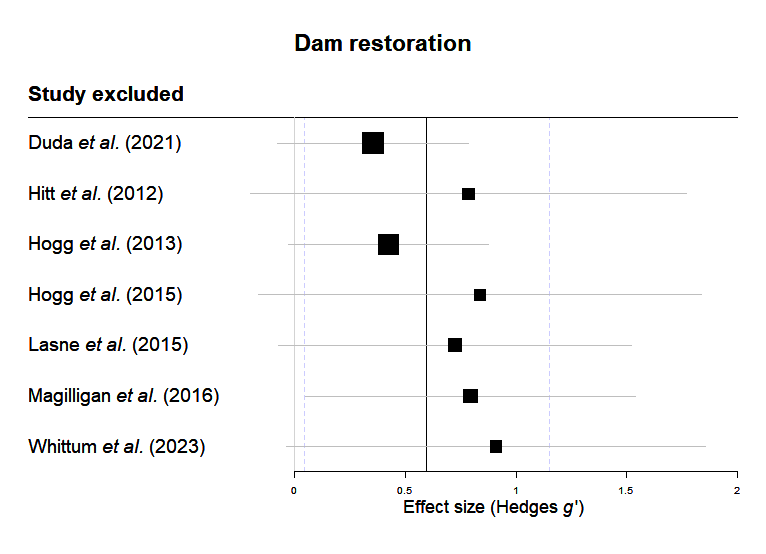
**

**Fig. S7.** Leave-one-out analysis for studies included in the meta-analysis that investigated dam removal. Each row represents the overall effect size with the corresponding study removed. Square sizes indicate the 95% confidence interval (CI), with larger squares representing smaller CIs and lower variability across studies. The solid vertical line denotes the overall effect size when all studies are included, while the dashed lines represent its CI. If the horizontal CI line of an effect size crosses zero after removing a study, the effect becomes non-significant.
